# Functional Studies of Missense TREM2 Mutations in Human Stem Cell-Derived Microglia

**DOI:** 10.1016/j.stemcr.2018.03.003

**Published:** 2018-03-29

**Authors:** Philip W. Brownjohn, James Smith, Ravi Solanki, Ebba Lohmann, Henry Houlden, John Hardy, Sabine Dietmann, Frederick J. Livesey

**Affiliations:** 1 The Gurdon Institute, ARUK Stem Cell Research Centre and Department of Biochemistry, University of Cambridge, Cambridge CB2 1QN, UK; 2 Department of Neurodegenerative Diseases, Hertie Institute for Clinical Brain Research, University of Tübingen, Tübingen 72076, Germany; 3 DZNE, German Center for Neurodegenerative Diseases, Tübingen 72076, Germany; 4 Department of Molecular Neuroscience, UCL Institute of Neurology, Queen Square, London WC1N 3BG, UK; 5 Wellcome Trust Medical Research Council Stem Cell Institute, University of Cambridge, Tennis Court Road, Cambridge CB2 1QR, UK

**Keywords:** dementia, microglia, TREM2, Nasu-Hakola disease, frontotemporal dementia, iPSC-microglia, neuroinflammation

## Abstract

The derivation of microglia from human stem cells provides systems for understanding microglial biology and enables functional studies of disease-causing mutations. We describe a robust method for the derivation of human microglia from stem cells, which are phenotypically and functionally comparable with primary microglia. We used stem cell-derived microglia to study the consequences of missense mutations in the microglial-expressed protein triggering receptor expressed on myeloid cells 2 (TREM2), which are causal for frontotemporal dementia-like syndrome and Nasu-Hakola disease. We find that mutant TREM2 accumulates in its immature form, does not undergo typical proteolysis, and is not trafficked to the plasma membrane. However, in the absence of plasma membrane TREM2, microglia differentiate normally, respond to stimulation with lipopolysaccharide, and are phagocytically competent. These data indicate that dementia-associated *TREM2* mutations have subtle effects on microglia biology, consistent with the adult onset of disease in individuals with these mutations.

## Highlights

• Microglia can be efficiently differentiated from human iPSCs
• iPSC microglia resemble human primary microglia transcriptionally and functionally
• Disease-causing mutations in *TREM2* affect receptor processing and expression
• *TREM2* mutant microglia differentiate, respond to pathogenic stimuli, and phagocytose

## Teaser

Brownjohn and colleagues report methods to generate microglia from induced pluripotent human stem cells, which they demonstrate are highly similar to cultured primary human microglia. Microglia differentiated from patient-derived stem cells carrying neurological disease-causing mutations in the *TREM2* receptor differentiate normally and respond appropriately to pathogenic stimuli, despite the absence of functional TREM2 receptor on the plasma membrane.

## Introduction

Microglia are brain-resident immune cells that perform key functions during nervous system development and homeostasis. After colonization and maturation in the developing CNS (Ginhoux et al., 2010, Ransohoff and Cardona, 2010), microglia shape synaptic connections between neurons through synapse pruning and provision of trophic support (Parkhurst et al., 2013, Schafer et al., 2012). As dynamic surveyors of the brain, microglia respond to damage- and pathogen-associated signals to maintain homeostasis (Koizumi et al., 2007, Nimmerjahn et al., 2005). In addition to developmental and homeostatic functions, it is now well established that microglia and associated neuroinflammation play roles in the progression of a number of neurodegenerative conditions (Perry et al., 2010, Ransohoff, 2016), with variants or mutations in microglia-expressed genes linked directly to disease in some cases (Malik et al., 2015, Paloneva et al., 2002, Sims et al., 2017). While microglial function and dysfunction are involved in many neurodegenerative conditions, their precise contributions to disease pathogenesis and progression are not well understood.

Avenues for the study of microglial biology in disease have primarily been limited to animal models and immortalized cell lines, both of which carry limitations in their ability to approximate primary human microglia. As more is understood about the developmental origin and unique identity of microglia, recent studies have attempted to circumvent this issue by deriving microglia from human induced pluripotent stem cells (iPSCs) in order to study human and cell-type-specific biology and disease (Abud et al., 2017, Douvaras et al., 2017, Haenseler et al., 2017, Muffat et al., 2016, Pandya et al., 2017, Takata et al., 2017). Here we describe and characterize a robust method for the derivation of microglia from human stem cells, which we then used to investigate the expressional and functional consequences of mutations in the microglia-expressed triggering receptor expressed on myeloid cells 2 (TREM2).

TREM2 is a transmembrane receptor expressed on cells of myeloid lineage, including osteoclasts and tissue-specific macrophages such as microglia (Colonna and Wang, 2016). Homozygous mutations in TREM2 or its intracellular signaling partner DAP12 are causal for Nasu-Hakola disease (NHD), which is associated with bone cysts and an early-onset dementia (Paloneva et al., 2000, Paloneva et al., 2002), while a frontotemporal dementia (FTD)-like syndrome without bone dysfunction has also been described in patients carrying certain *TREM2* mutations (Chouery et al., 2008, Giraldo et al., 2013, Guerreiro et al., 2013b). The recent discovery that heterozygous coding variants in *TREM2* confer an increased risk of Alzheimer's disease (AD) (Guerreiro et al., 2013a, Jin et al., 2014, Jonsson et al., 2013) has reignited interest in understanding the role of this receptor in microglial function.

While the endogenous ligand has not been confirmed, *in vitro* studies have demonstrated binding of TREM2 to lipoprotein, apolipoprotein, and pathogen- and damage-associated ligands (Atagi et al., 2015, Bailey et al., 2015, Daws et al., 2003, Yeh et al., 2016). FTD-like and NHD mutations in *TREM2* are described as loss-of-function mutations, as they result in reduced cell surface expression and ligand binding (Kleinberger et al., 2014, Kober et al., 2016, Park et al., 2015), while AD-associated variants are thought to reduce the affinity of TREM2 for its ligands (Kober et al., 2016, Yeh et al., 2016). Extensive studies have ascribed a number of functions to TREM2, including regulation of phagocytosis (Hsieh et al., 2009, Kleinberger et al., 2014, Takahashi et al., 2005), cytokine release (Hamerman et al., 2006, Turnbull et al., 2006), chemotaxis (Mazaheri et al., 2017), and cell survival (Wang et al., 2015). While murine models of neurodegenerative disease indicate that loss or dysfunction of TREM2 signaling impacts upon microglial function and disease progression (Ulrich et al., 2014, Yuan et al., 2016), the precise role of TREM2 in microglial biology and the consequences of its dysregulation in neurodegenerative disease pathogenesis remain to be determined. Therefore, we used our method for generating human microglia to study the expression, cellular localization, and function of TREM2 in microglia differentiated from iPSCs derived from individuals carrying *TREM2* mutations causal for FTD-like syndrome and NHD.

## Results

### Human Stem Cell-Derived Microglia Phenotypically Resemble Primary Microglia

Microglia differ from other adult tissue-resident macrophages in two key ways; firstly, their yolk-sac-derived progenitors arise early in development from a program of primitive hematopoiesis rather than the later definitive hematopoiesis that replaces many tissue-resident macrophages in the developed adult (Ginhoux et al., 2010, Ginhoux et al., 2013, Kierdorf et al., 2013, Schulz et al., 2012), and secondly their transcriptome, reflective of the brain-specific roles they perform, is distinct from other myeloid cells (Bennett et al., 2016, Butovsky et al., 2014, Hickman et al., 2013).

**Figure 1:**
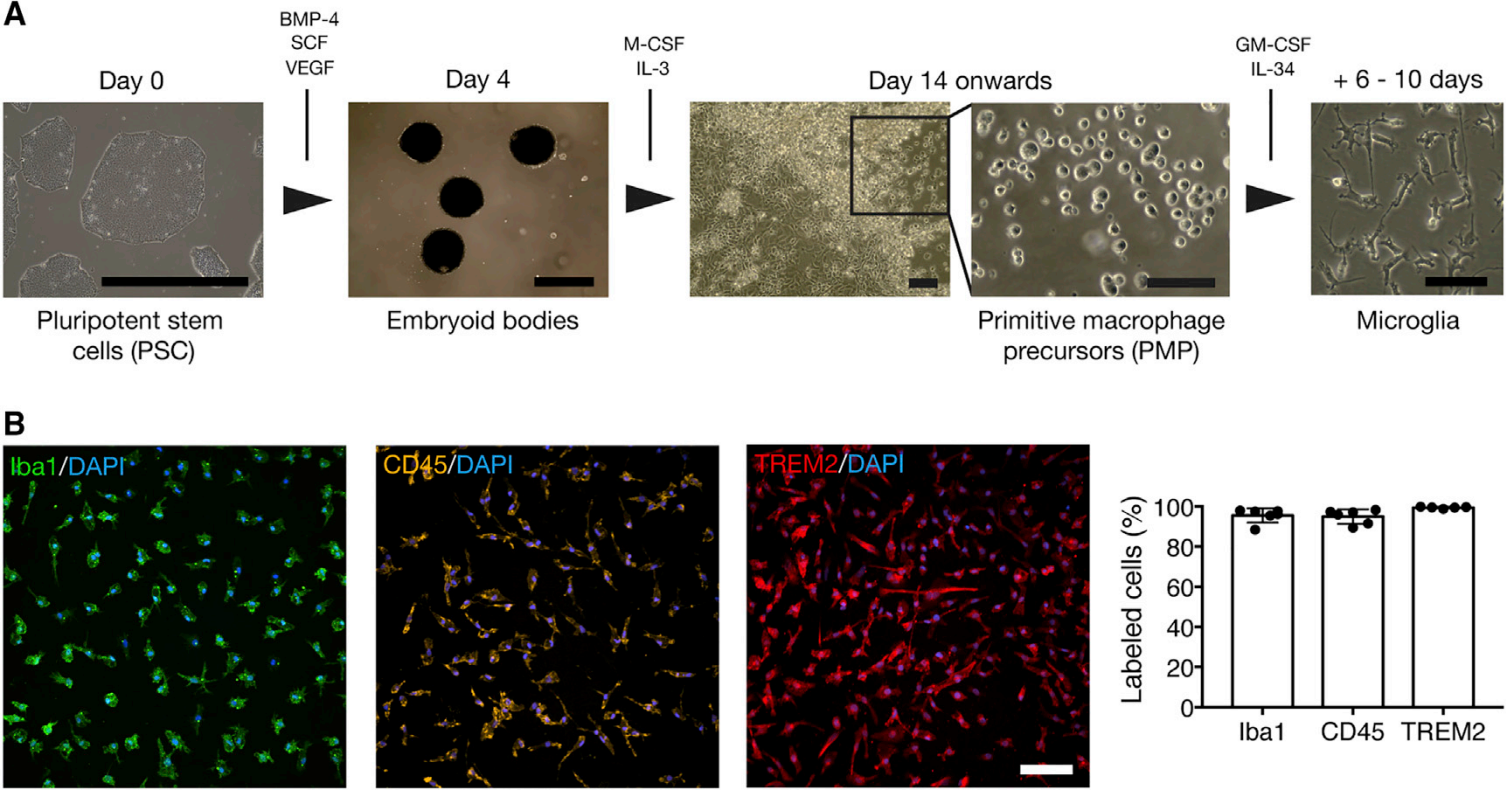
An Efficient Protocol for the Generation of Microglia from Pluripotent Stem Cells (A) PSCs are differentiated to microglia via embryoid bodies and PMPs. PMPs are produced continuously in culture and are terminally differentiated into microglia when required. (B) A high proportion of stem cell-derived microglia express the microglial/macrophage markers Iba1, CD45, and TREM2. Scale bars represent 100 μm, except PSC and embryoid bodies (1 mm). n = 5–6 biological replicates. Error bars represent SDs.

As a starting point for the differentiation of microglia, we followed an established method for the derivation of primitive macrophage precursors (PMPs) from human pluripotent stem cells (PSCs) (Karlsson et al., 2008, van Wilgenburg et al., 2013). It has recently been shown that these precursors are produced in a Myb-independent manner, in a pathway closely recapitulating primitive hematopoiesis (Buchrieser et al., 2017). Two to three weeks after the initiation of differentiation, PMPs are produced continuously in suspension, and can be harvested for further maturation. The PMP generation phase can continue indefinitely and is particularly efficient: in the longest ongoing differentiation in this study, one million PSCs produced between 23 and 52 million PMPs in seven PSC lines over 80 days, similar to previously reported PMP yields using the same method (Haenseler et al., 2017, van Wilgenburg et al., 2013) and microglia yields using a recently described alternative method (Abud et al., 2017). Using complete RPMI1640 containing a combination of granulocyte macrophage colony-stimulating factor (GM-CSF) and interleukin-34 (IL-34) (Ohgidani et al., 2014), we differentiated PMPs over 6–10 days to produce monocultures that morphologically resemble microglia (Figure 1A). Analysis of the proportion of these cells expressing canonical macrophage/microglia markers indicates that this protocol has a high level of efficiency across genetic backgrounds, producing cells 95.6% ± 3.6% positive for Iba1 (mean ± SD, n = 6), 95.0% ± 3.6% positive for CD45 (mean ± SD, n = 6), and 99.5% ± 0.4% positive for TREM2 (mean ± SD, n = 5) (Figure 1B).

**Figure 2:**
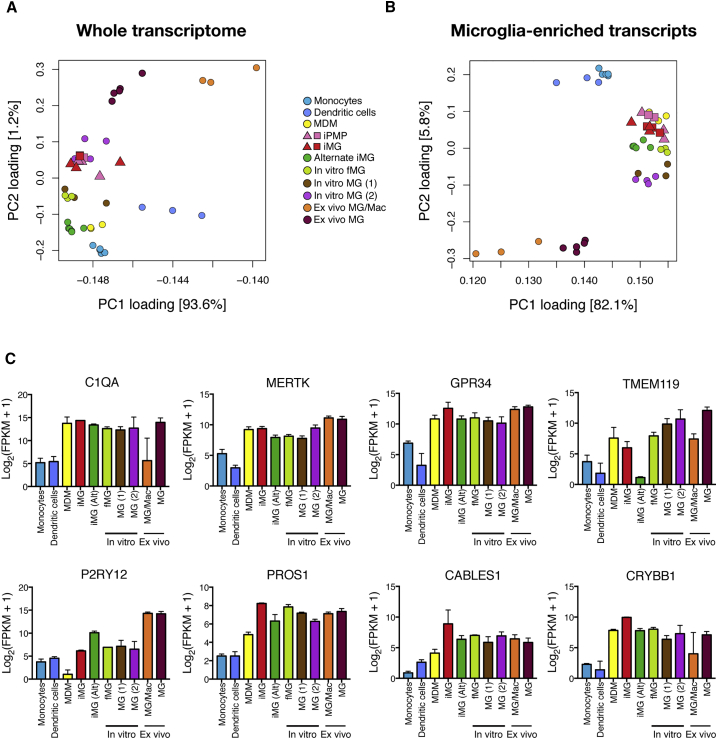
The Transcriptome of Human Stem Cell-Derived Microglia Is Similar to Primary Microglia (A) At the whole-transcriptome level, iPSC-derived primitive macrophage precursors (iPMPs) and iPSC-derived microglia (iMG) cluster with primary microglia cultured *in vitro* (In vitro MG (2)). (B) When compared using a subset of genes enriched in murine microglia over other CNS myeloid cells (Bennett et al., 2016), iMG and iPMPs again cluster with primary microglia cultured *in vitro* (In vitro fetal MG [fMG], In vitro MG (1), and In vitro MG (2)), and additionally with an alternative iMG method (Alternate iMG; Abud et al., 2017) and monocyte-derived macrophages (MDM). (C) FPKM (fragments per kilobase million) counts of “microglia signature” genes indicate comparable levels of expression in iMG compared with *in vitro* and *ex vivo* primary microglia. Monocytes, CD14+/CD16− monocytes (Abud et al., 2017); MDM, monocyte-derived macrophages (Zhang et al., 2015); iPMPs, iPSC-derived primitive macrophage precursors (this study); iMG, iPSC-derived microglia (this study); iMG (alternate), iPSC-microglia derived using an alternative method (Abud et al., 2017); In vitro fMG, *in vitro* fetal microglia (Abud et al., 2017); In vitro MG (1), *in vitro* microglia (Abud et al., 2017); In vitro MG (2), *in vitro* microglia (Gosselin et al., 2017); Ex vivo MG/Mac, primary sorted CNS CD45+ microglia/macrophages (Zhang et al., 2016); Ex vivo MG, primary sorted microglia (Gosselin et al., 2017). For iPMPs and iMG, n = 3 independent differentiations of 2 genetic backgrounds (n = 5 iPMPs, n = 6 iMG). In (A) and (B), genetic backgrounds of iPMPs and iMG are distinguished by square and triangle symbols. For (C), iMG FPKM values were averaged across differentiations to give values for each genetic background (n = 2). Error bars represent SDs.

To investigate the transcriptional identity of our stem cell-derived microglia in the context of the wider myeloid family, we used RNA sequencing (RNA-seq) to compare the transcriptome of these microglia with a number of published datasets: primary *ex vivo* CNS CD45+ microglia/macrophages (Zhang et al., 2016) and microglia (Gosselin et al., 2017), primary microglia and fetal microglia cultured *in vitro* for 7–10 days (Abud et al., 2017, Gosselin et al., 2017), myeloid cells of alternate lineages (monocyte-derived macrophages (Zhang et al., 2015), CD14+/CD16− monocytes (Abud et al., 2017) and dendritic cells (Abud et al., 2017)), and iPSC-microglia derived using an alternative method (Abud et al., 2017) (Figure 2). The recently published dataset from Gosselin et al. (2017) is a particularly useful addition to our understanding of microglial identity, as it compares the transcriptomes of freshly isolated *ex vivo* microglia with the same cells cultured *in vitro* for 7–10 days, allowing assessment of how culture environment alters the transcriptome. PMPs and microglia derived from three independent differentiations from two genetic backgrounds were harvested for RNA-seq analysis, in order to assess reproducibility of the differentiation process both within and between genetic backgrounds.

At the whole-transcriptome level, PMPs and microglia generated by the method reported here most closely resemble cultured primary microglia (Figure 2A). Due to a lack of unique surface markers, it has historically been difficult to distinguish microglia from other macrophages and cells of myeloid lineage. It is only recently that a distinct transcriptomic profile of microglia has emerged (Bennett et al., 2016, Butovsky et al., 2014, Hickman et al., 2013). Using a subset of genes from a recent study that were enriched in expression in murine microglia compared with other CNS myeloid cells (Table S1) (Bennett et al., 2016), we again observed that microglia derived from iPSCs using this method cluster most closely with cultured primary microglia, in addition to monocyte-derived macrophages and iPSC-microglia derived using an alternative method (Figure 2B). It is notable that, using this approach, there are three obvious clusters of cell type: *in vitro* cultured microglia/macrophages regardless of origin group together, flanked by a cluster of *ex vivo* microglia and microglia/macrophages and a separate cluster of primary *ex vivo* myeloid cells (dendritic cells and CD14+/CD16− monocytes).

Closer analysis of the expression of microglia “signature” genes (Bennett et al., 2016, Butovsky et al., 2014, Hickman et al., 2013) indicates that stem cell-derived microglia express many of these enriched microglial transcripts at comparable levels with primary *ex vivo* and *in vitro* human microglia (Figure 2C). In sum, these data indicate a close similarity of stem cell-derived microglia with primary microglia at the whole-transcriptome and microglia-specific transcript level.

### Functional Characterization of Human Stem Cell-Derived Microglia

**Figure 3:**
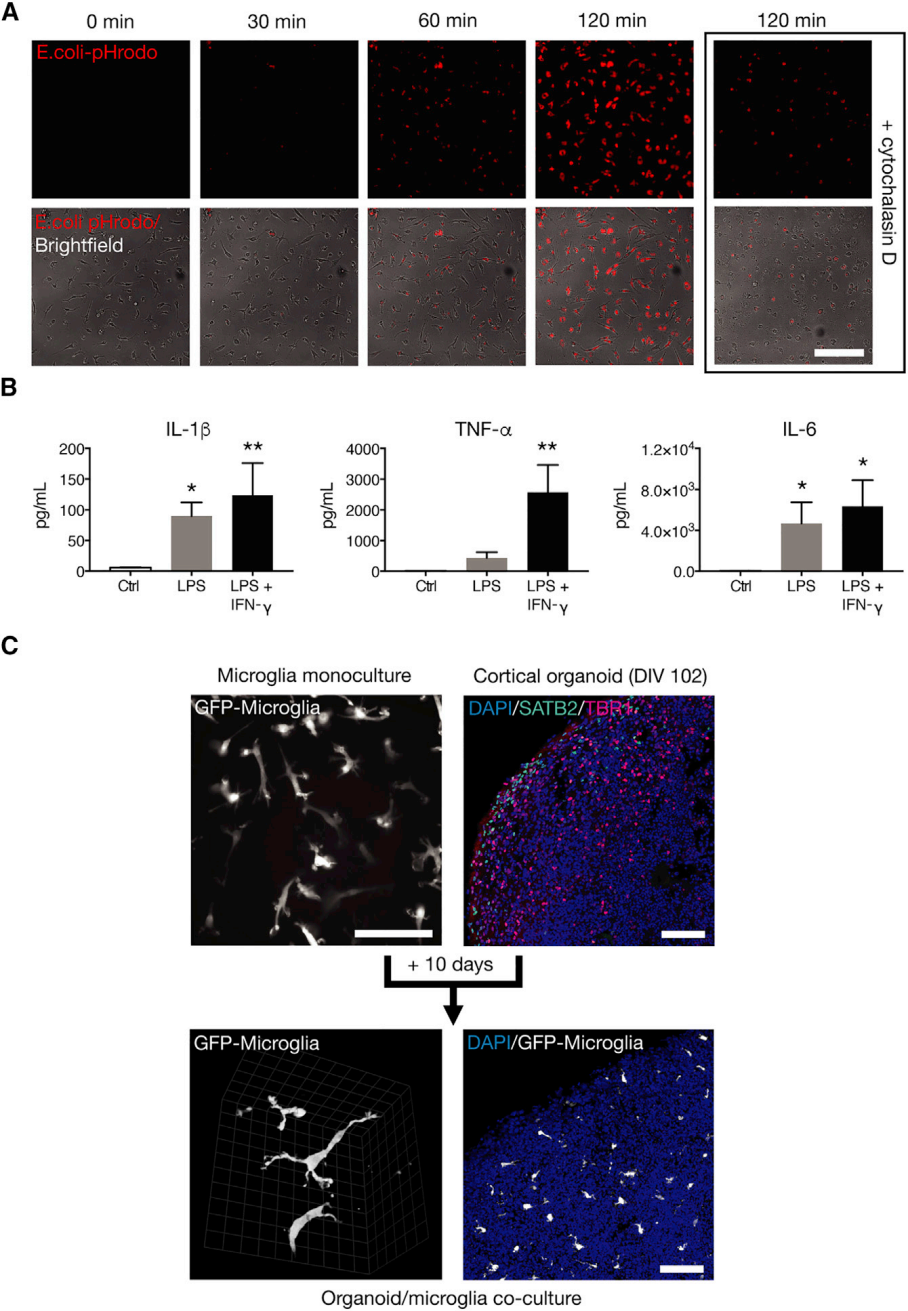
Stem Cell-Derived Microglia Are Functionally Similar to Primary Microglia (A) Microglia efficiently phagocytose pHrodo-*E. coli*, in a process sensitive to cytochalasin D. (B) Upon exposure to 100 ng/mL LPS, microglia secrete pro-inflammatory cytokines; an effect augmented by interferon γ (IFN-γ). (C) Microglia migrate into preformed cortical organoids. Upon migration, microglia tessellate throughout organoids and assume a pronounced ramified morphology, which is demonstrated by live 2-photon imaging of organoid/microglia co-cultures. Scale bars represent 200 μm (A) and 100 μm (C), while scale grid markings at high magnification represent 12.4 μm. In (B), n = 3 biological replicates; ^∗^ p < 0.05, ^∗∗^ p < 0.01 treatment versus control; Dunnett's *post hoc* test. Error bars represent SDs.

Microglia are professional phagocytes, able to clear pathogens and cellular debris. Using live imaging, we found that stem cell-derived microglia efficiently phagocytose bacterial particles, a process attenuated by the actin polymerization inhibitor cytochalasin D (Figure 3A). Microglia express pattern recognition receptors such as Toll-like receptors (TLRs) that mediate responses to pathogenic stimuli. Exposure of microglia to the TLR4 ligand lipopolysaccharide (LPS) alone, or in combination with the immunomodulatory cytokine interferon γ, resulted in an upregulation of the pro-inflammatory cytokines IL-1β (F_(2, 6)_ = 10.26, p = 0.0116), tumor necrosis factor alpha (TNF-α) (F_(2, 6)_ = 20.66, p = 0.0020), and IL-6 (F_(2, 6)_ = 8.848, p = 0.0162) over 24 hr (Figure 3B), confirming that stem cell-derived microglia respond appropriately to pathogenic stimuli.

While maintenance of microglia in monoculture is useful for reductionist studies of defined functions, more complex co-culture models of microglia with neurons have the potential to enable studies of more complex biology, including microglia migration, interactions with neurons, and homing to areas of neuronal injury. To explore the ability of stem cell-derived microglia to invade developing neural tissues and migrate within them, we added microglia to preformed 3D cortical organoids that were cultured in excess of 100 days *in vitro*. Under these conditions, we found that stem cell-derived microglia migrated from the surface deeply into organoids (Figure 3C), and, upon integration, assumed a pronounced ramified morphology, surviving in those environments for at least several weeks in the absence of continued supplementation of the medium with colony-stimulating factors.

### Expression of Mutant TREM2 in Stem Cell-Derived Microglia

To study the function and dysfunction of TREM2 in microglia, we investigated the most severe form of TREM2 disruption in humans, that of the missense mutations causing FTD-like syndrome and NHD. We obtained fibroblasts from a patient homozygous for the *TREM2* T66M mutation, associated with an FTD-like condition (Guerreiro et al., 2013b) (T66M/T66M), two unaffected family members carrying a single copy of the T66M mutation (wt/T66M), and a patient homozygous for the recently described *TREM2* W50C mutation causal for NHD (Dardiotis et al., 2017) (W50C/W50C), and reprogrammed them to iPSCs. After characterizing iPSC lines (Figures S1–S3), we confirmed their capacity to differentiate into microglia by expression of a number of microglia-enriched genes (Figure S4).

**Figure 4:**
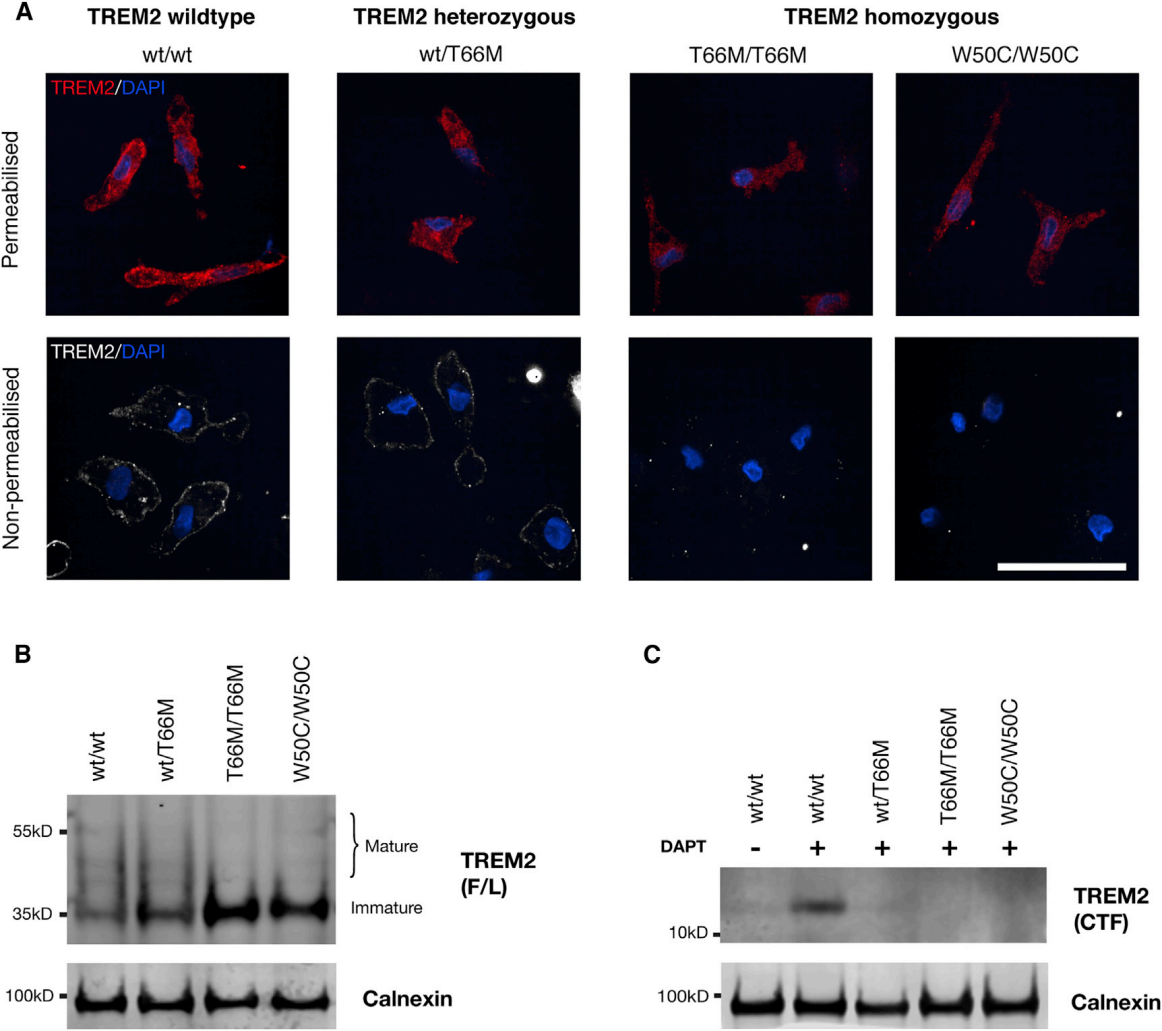
The TREM2 Receptor Is Mislocalized and Aberrantly Processed in *TREM2* Mutant Microglia (A) Staining for the TREM2 receptor with an N-terminally directed primary antibody reveals expression of TREM2 protein in microglia from *TREM2* wild-type and mutant microglia when cells are permeabilized. Upon omission of permeabilization, TREM2 receptor surface staining is only detected in *TREM2* wild-type and heterozygous mutant microglia, and is absent in microglia derived from homozygous mutant backgrounds. (B) Probing of whole-cell lysates reveals expression of immature and mature forms of the full-length (F/L) TREM2 receptor in wild-type microglia. Mutations in *TREM2* cause a gene-dosage-dependent accumulation of immature TREM2, and a reduction in mature forms of TREM2. (C) Probing with a C-terminally directed antibody reveals a weak band corresponding to the CTF of TREM2 in wild-type microglia, indicating efficient turnover of the CTF. Overnight treatment with DAPT (10 μM) results in accumulation of the TREM2-CTF in *TREM2* wild-type microglia, a barely detectable accumulation in heterozygous mutant microglia and no detectable accumulation in homozygous mutant microglia. Scale bar represents 50 μm.

In heterologous cell systems, it has been demonstrated that missense mutations in the *TREM2* gene disrupt intracellular trafficking and protein maturation of TREM2, ultimately reducing functional cell surface expression and ectodomain shedding (Kleinberger et al., 2014, Kober et al., 2016, Park et al., 2015). We sought to determine if cell surface expression, maturation, and proteolysis of TREM2 was impaired in microglia harboring missense mutations in *TREM2* (Figure 4). Using an N-terminal antibody against the extracellular ectodomain of TREM2, we performed immunofluorescence with or without permeabilization of the plasma membrane (Figure 4A). With permeabilization, TREM2 protein was detected in wild-type microglia, as well as microglia from heterozygous (wt/T66M) and homozygous (T66M/T66M and W50C/W50C) *TREM2* mutant backgrounds. In the absence of permeabilization, plasma membrane TREM2 was only detected in wild-type and heterozygous (wt/T66M) *TREM2* backgrounds, indicating a loss of functional receptor surface expression in homozygous *TREM2* mutant microglia.

Western blot analysis of whole-cell lysates indicated that *TREM2* wild-type microglia express both mature and immature forms of TREM2, but there is a gene dosage-dependent reduction in mature forms and a concomitant increase in immature forms of TREM2 in mutant microglia (Figure 4B), indicating mutation-dependent effects on protein maturation. Full-length TREM2 undergoes regulated intramembrane proteolysis (RIP) by the metalloproteases ADAM17 and ADAM10 (Feuerbach et al., 2017, Kleinberger et al., 2014), which releases a soluble TREM2 fragment and leaves a membrane-bound C-terminal fragment (CTF) that is a substrate for the γ-secretase complex (Wunderlich et al., 2013). To determine the effects of missense mutations on TREM2 RIP, we probed whole-cell lysates with a C-terminally directed TREM2 protein antibody. In the steady state, TREM2-CTF is barely detectable in wild-type microglia, indicating rapid turnover by γ-secretase (Figure 4C). Following treatment with a γ-secretase inhibitor (DAPT), the levels of TREM2 CTF increase in *TREM2* wild-type microglia, but are barely detectable in heterozygous (wt/T66M) and not detectable in homozygous (T66M/T66M and W50C/W50C) *TREM2* mutant microglia, indicating reduced proteolysis of TREM2, most likely due to reduced cell surface expression and shedding of soluble TREM2.

We conclude that the consequences of both *TREM2* missense mutations are complex: there is no detectable TREM2 on the plasma membrane, a reduction in maturation of the TREM2 protein, and reduced generation of the CTF of TREM2. While FTD-like and NHD mutations have been described as loss-of-function mutations, they are not all alike. Nonsense mutations such as Q33X result in a truncated protein, whereas missense mutations such as T66M (and possibly W50C (Dardiotis et al., 2017)) result in aberrant trafficking and either loss of expression or expression of misfolded and non-functional protein on the cell surface (Kleinberger et al., 2014, Kober et al., 2016). In that respect, missense mutations in *TREM2* may not be the equivalent of simple loss-of-function mutations, and may have complex downstream effects on interacting partners and intracellular trafficking machinery.

### TREM2 Mutant Microglia Respond Appropriately to Pathogenic Challenge

**Figure 5:**
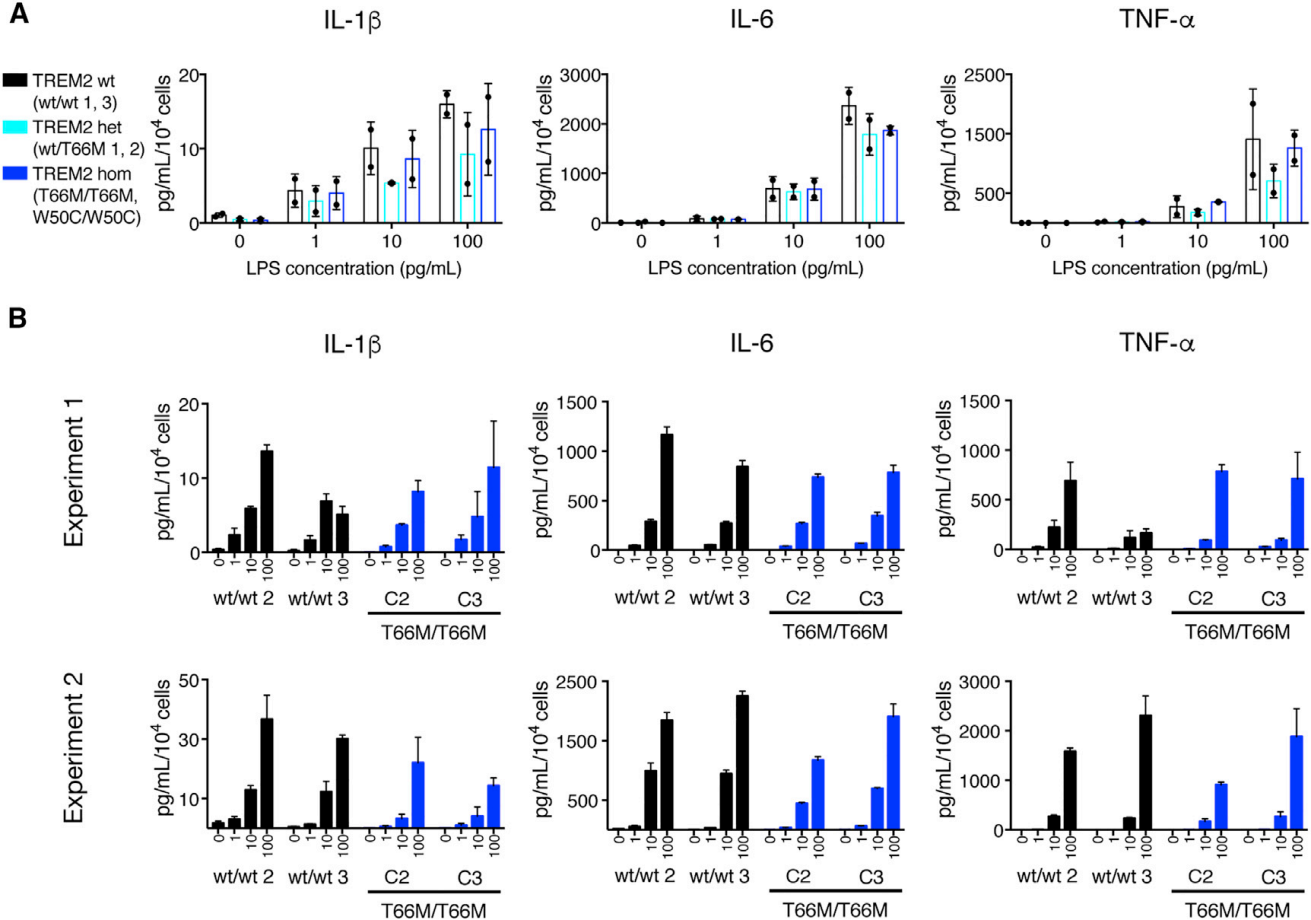
Microglia Harboring TREM2 Mutations Respond Appropriately to LPS Challenge (A) Upon exposure to LPS, microglia from *TREM2* wild-type and mutant backgrounds release pro-inflammatory cytokines in a dose-dependent manner. (B) Further experiments in two additional clones from patient T66M/T66M confirm a similar response to LPS challenge across all clones of this genotype. Results in (A) are an average of all data from two independently performed treatments, n = 2 biological replicates per genotype and error bars represent SDs. Error bars in (B) represent SE of 2–3 technical well replicates, and x-axis values are LPS concentration in pg/mL.

It has been reported that TREM2 modulates inflammatory responses to pathogenic stimuli (Ito and Hamerman, 2012, Turnbull et al., 2006). We exposed microglia from *TREM2* wild-type and mutant backgrounds to LPS, and measured the release of IL-1β, IL-6, and TNF-α in the extracellular medium after 6 hr (Figure 5A). Two-way ANOVA revealed that, while LPS concentration had a significant effect on the release of IL-1β (F_(3, 12)_ = 16.94, p = 0.0001), IL-6 (F_(3, 12)_ = 133.5, p = < 0.0001), and TNF-α (F_(3, 12)_ = 21.73, p = < 0.0001), there was no significant effect of genotype on cytokine release, and no significant interaction between LPS concentration and genotype. To assess whether clonal differences in reprogrammed iPSC lines might contribute to the observed lack of effect of *TREM2* mutations on response to LPS, we performed further experiments on microglia differentiated from two additional clones of T66M/T66M mutant iPSCs. Again, we found that *TREM2* mutant microglia released similar levels of cytokines to *TREM2* wild-type microglia in response to LPS (Figure 5B). This indicates that *TREM2* mutant microglia have a normal physiological response to pathological stimuli in this system.

### TREM2 Mutant Microglia Are Phagocytically Competent

**Figure 6:**
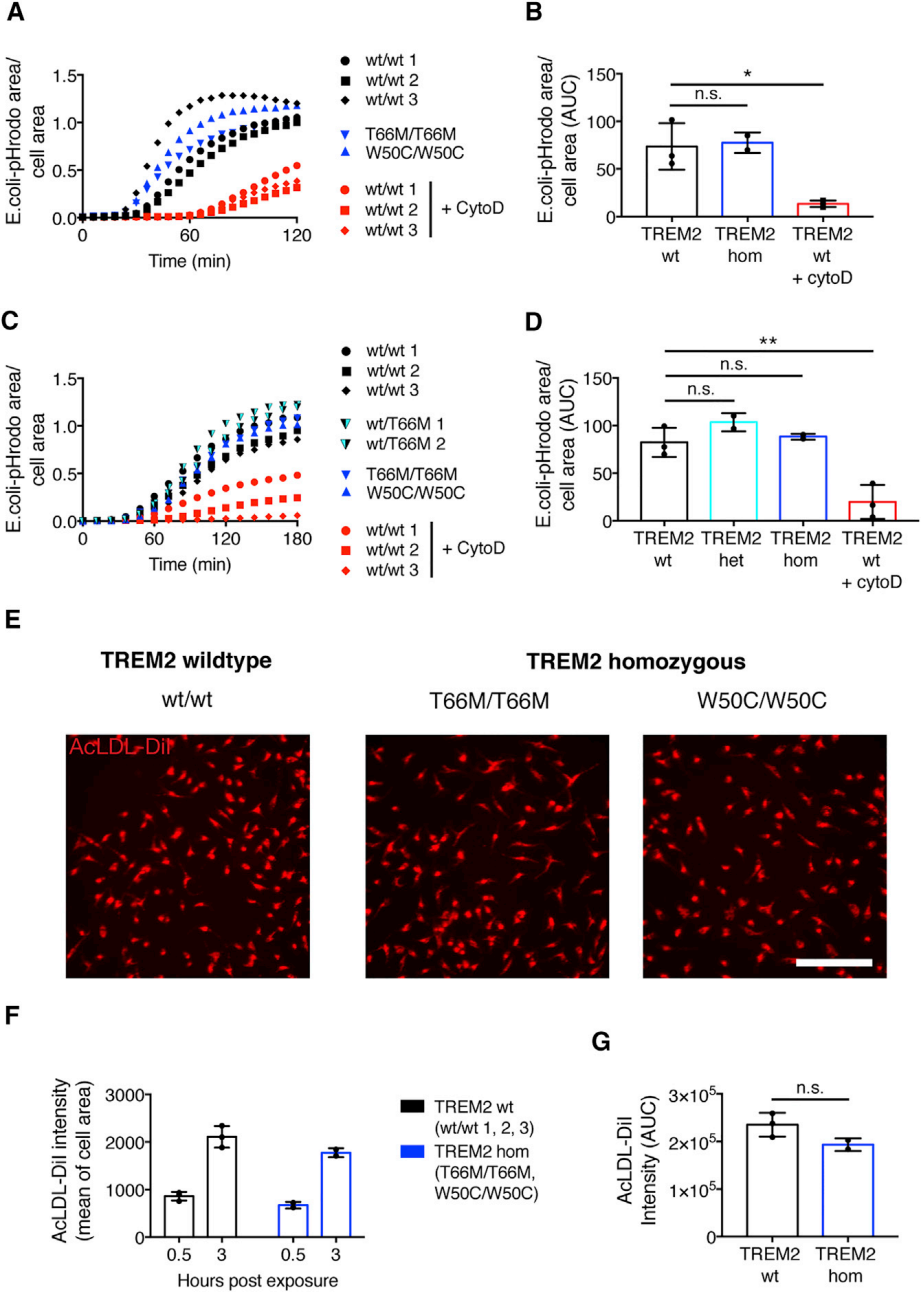
Microglia from *TREM2* Mutant Backgrounds Are Phagocytically Competent (A and B) Homozygous *TREM2* mutant microglia phagocytose pHrodo-*E. coli* with a similar efficiency and capacity to microglia from a wild-type background. In contrast, cytochalasin D significantly attenuates pHrodo-*E. coli* uptake. (C and D) Similarly, after serum starvation, *TREM2* mutant microglia phagocytose *E. coli* as efficiently as *TREM2* wild-type microglia. (E–G) *TREM2* mutant microglia internalize AcLDL almost as efficiently as *TREM2* wild-type microglia. Scale bar represents 200 μm. All data from two (C, D, and E–G) or three (A and B) independent experiments were averaged to produce values for each genotype. n = 3 (*TREM2* wild-type-untreated; *TREM2* wild-type cytochalasin D-treated) or 2 (*TREM2* heterozygous; *TREM2* homozygous) biological replicates. ^∗^p < 0.05, ^∗∗^p < 0.01 versus untreated *TREM2* wild-type microglia, Dunnett's *post hoc* test. n.s., not significant. Error bars represent SDs.

As TREM2 has a proposed role in phagocytosis, we sought to determine if missense mutations in this gene had a significant effect on microglia clearance of bacterial particles. Live imaging over 2 hr with pHrodo-*E. coli* particles revealed that microglia from wild-type *TREM2* backgrounds efficiently phagocytose bioparticles (Figures 6A and 6B). While one-way ANOVA of the 2 hr area under curve (AUC) indicated differences between treatment and genotype groups (F_(2, 5)_ = 13.42, p = 0.0098), Dunnett's *post hoc* testing revealed that *TREM2* wild-type microglia phagocytosis was significantly inhibited by cytochalasin D (p = 0.0111, compared with untreated *TREM2* wild-type microglia); however, microglia differentiated from T66M/T66M and W50C/W50C *TREM2* mutant backgrounds phagocytosed bioparticles as efficiently as microglia from wild-type *TREM2* backgrounds (p = 0.9519, compared with untreated *TREM2* wild-type microglia).

Our microglia medium contains serum, which is known to opsonize and enhance phagocytic mechanisms. To investigate whether serum could mask potential deficits in phagocytosis, we repeated *E. coli*-uptake experiments in the absence of serum, following overnight serum starvation (Figures 6C and 6D). In serum-starved conditions, microglia again phagocytosed *E. coil* particles, although notably at a slower rate than in serum-containing conditions. One-way ANOVA of the 3 hr AUC indicated a difference between groups (F_(3, 6)_ = 18.03, p = 0.0021), which Dunnett's *post hoc* testing revealed was driven by a reduced particle uptake in cytochalasin D-treated *TREM2* wild-type microglia (p = 0.0042, compared with untreated *TREM2* wild-type microglia); however, no differences in uptake comparing heterozygous or homozygous *TREM2* mutant microglia with *TREM2* wild-type microglia (p = 0.3212 and p = 0.9361, respectively, compared with untreated wild-type *TREM2* microglia).

In addition to its proposed role as a phagocytosis receptor, TREM2 has been demonstrated to be an uptake receptor for number of proteins, including acetylated LDL (AcLDL) (Yeh et al., 2016). To assess the uptake of AcLDL in microglia, and the consequences of *TREM2* mutations, microglia from *TREM2* wild-type and homozygous mutant backgrounds were exposed to labeled AcLDL and assessed 30 min and 3 hr post exposure for internalized AcLDL (Figures 6E–6G). Two-way ANOVA revealed a statistically significant effect of genotype (F_(1, 6)_ = 7.28, p = 0.0357) on the uptake of AcLDL; however, there was no interaction between genotype and time point (F_(1, 6)_ = 0.5492, p = 0.4866), and the effect between genotypes was not significant at either time point alone (p = 0.3848 and p = 0.0995 at 30 min and 3 hr, respectively), or when compared using the 3 hr uptake AUC (t_(3)_ = 2.104, p = 0.1261), indicating a minor impairment of AcLDL uptake in *TREM2* homozygous mutant microglia, consistent with the presence of a number of scavenger receptors for AcLDL in addition to TREM2 (Canton et al., 2013).

## Discussion

We describe the derivation and characterization of microglia from human stem cells, which transcriptionally and functionally resemble primary microglia. Using these cells as a model for microglial biology, we probed the consequences of *TREM2* mutations using microglia derived from patients carrying missense mutations in *TREM2* causative for the neurodegenerative conditions FTD-like syndrome and NHD. We found that TREM2 is aberrantly processed in microglia from *TREM2* mutant backgrounds, accumulating in its immature form, and, furthermore, is not trafficked appropriately to the cell membrane in homozygous mutant microglia. Unexpectedly, despite dysregulation of TREM2 protein expression, mutant microglia differentiate properly, effectively respond to pathogenic stimuli, and phagocytose appropriately.

Microglia originate from PMPs that invade the developing CNS and mature into microglia (Ginhoux et al., 2010, Ginhoux et al., 2013). To reproduce the developmental origin of microglia, we began with a protocol for the generation of PMPs (Karlsson et al., 2008, van Wilgenburg et al., 2013). These precursors are not dependent on the transcription factor Myb (Buchrieser et al., 2017), and are therefore closer in lineage to yolk-sac-derived precursors of microglia than Myb-dependent precursors of the definitive hematopoiesis pathway (Schulz et al., 2012). Using IL-34, which is produced by neurons in the developing CNS, and is essential for microglial development and survival (Wang et al., 2012), together with GM-CSF, we matured these macrophage precursors into microglia. The entire protocol is rapid and efficient, producing microglia that express a transcriptional and functional signature comparable with primary human microglia within 4 weeks. Over the course of this study, a number of microglia differentiation protocols were reported (Abud et al., 2017, Douvaras et al., 2017, Haenseler et al., 2017, Muffat et al., 2016, Pandya et al., 2017). This method compares favorably with these recent studies, with a simple, sorting-free protocol that yields comparable numbers of microglia over a similar timescale (Abud et al., 2017, Haenseler et al., 2017), and a comparable transcriptome with the microglia described by Abud et al. (2017), which relies on a much more complex differentiation method.

The transcriptional signature associated with microglial identity is rapidly lost upon removal from the neural environment, making isolated culture of microglia challenging (Bohlen et al., 2017, Gosselin et al., 2017). While efforts have been made to understand the environmental cues driving microglial identity (Bohlen et al., 2017, Butovsky et al., 2014), there are still gaps in our knowledge regarding how medium composition can influence the transcriptome, as further demonstrated in this study, which indicates a clear separation between freshly isolated *ex vivo* and *in vitro* cultured microglia, even between samples from the same individual (Gosselin et al., 2017). Expression of the microglia-enriched gene *TMEM119,* for example, is particularly strongly downregulated in microglia removed from the CNS (Gosselin et al., 2017). The presence of serum in microglial maintenance medium has been reported to induce expression of this gene (Douvaras et al., 2017), however, perhaps explaining why some *TMEM119* expression is observed in our microglia, but not those derived in another study that used defined conditions (Abud et al., 2017). While the use of serum has some drawbacks, including introducing potential variability into differentiation efficiency, we have shown, both transcriptionally and by expression of the macrophage/microglial markers Iba1, CD45, and TREM2, that our differentiation protocol is consistent in efficiency across genetic backgrounds, indicating an acceptable level of variability for studying microglial biology and pathophysiology.

Missense mutations in *TREM2* are thought to affect protein structure, leading to trafficking defects and reduced cell surface expression. We have determined that the FTD-like mutation T66M results in protein mis-processing and reduced surface expression of TREM2 in stem cell-derived microglia, as was previously demonstrated in heterologous cell systems (Kleinberger et al., 2014, Kober et al., 2016, Park et al., 2015). Likewise, the recently identified NHD mutation W50C results in similar mis-regulation, as was predicted by structural modeling (Dardiotis et al., 2017). As TREM2 is postulated to regulate the innate immune system, and is thought to directly modulate phagocytosis and responses to pathogens, we chose to investigate these functions in patient-derived microglia from these *TREM2* mutant backgrounds, and found, surprisingly, that these functions remained intact even in presumed loss-of-function *TREM2* backgrounds.

When TREM2 is overexpressed in non-phagocytic cells, it confers phagocytic activity (Kleinberger et al., 2014, N'Diaye et al., 2009). Likewise, in murine macrophages and microglia, genetic disruption of *TREM2* has been reported to impair the uptake of synthetic material, bacteria, and apoptotic neurons (Hsieh et al., 2009, Kleinberger et al., 2017, Kleinberger et al., 2014, N'Diaye et al., 2009, Takahashi et al., 2005), although this has not always been observed (Wang et al., 2015). It is perhaps surprising that we did not observe impaired phagocytosis in *TREM2* mutant microglia, although the impact of TREM2 disruption on phagocytosis has not previously been demonstrated in human phagocytes expressing physiological levels of TREM2 protein. It is possible that the wide range of phagocytic receptors in microglia (Sierra et al., 2013) acts to compensate for a loss of TREM2, thus masking any potential impairment. Similarly, for the uptake of AcLDL, which we demonstrated is only mildly impaired in microglia derived from *TREM2* mutant backgrounds compared with wild-type backgrounds, there are many receptors that potentially internalize ligands of TREM2, making it difficult to establish a deficit due to loss of function of TREM2 alone. TREM2 has also been demonstrated as a modulator of TLR signaling (Ito and Hamerman, 2012, Turnbull et al., 2006, Wang et al., 2015). While we did not observe differential responses of *TREM2* mutant microglia to the TLR4 ligand LPS, it is known that effects of TREM2 on innate immune responses are variable, and depend upon ligand, cell type, and model system (Ito and Hamerman, 2012, Kang et al., 2018, Turnbull et al., 2006). It is possible that TREM2 has more subtle or context-dependent roles in microglial immune regulation that will require more complex culture conditions to investigate (Keren-Shaul et al., 2017, Krasemann et al., 2017).

In summary, this report details the utility of a simple and effective method for generating microglia from human stem cells. Microglia differentiated from patients carrying missense mutations in *TREM2* that are causal for FTD-like syndrome and NHD accumulate immature TREM2 protein, and do not express functional TREM2 on the cell surface. Despite aberrant TREM2 processing, microglia from mutant *TREM2* backgrounds respond appropriately to challenge with pathogens, releasing similar levels of inflammatory cytokines and phagocytosing as efficiently as *TREM2* wild-type microglia. These results suggest a complex and subtle effect of missense *TREM2* mutations on microglial function that may take some time to manifest in the clinical symptoms, in line with the adult onset of dementia in FTD-like syndrome and NHD.

## Experimental Procedures

See Supplemental Information for full details of all methods used.

### Microglia Differentiation

Details of PSC lines used in this study can be found in Supplemental Experimental Procedures. *TREM2* mutant lines were generated from fibroblasts derived from skin punch biopsies obtained following informed consent, after approval from the ethics committee of the Istanbul Faculty of Medicine, Istanbul University, or the joint research ethics committee of the National Hospital for Neurology and Neurosurgery and the Institute of Neurology, UCL. Microglia were differentiated from PSCs via embryoid bodies and PMPs (Karlsson et al., 2008, van Wilgenburg et al., 2013). In brief, at least 2 days after last passaging, PSCs (cultured feeder-free in E8) were passaged to single cells with TrypLE Express (Gibco), and plated at 10,000 per well in 96-well ultra-low attachment plates (Corning) in 100 μL embryoid body medium (10 μM ROCK inhibitor, 50 ng/mL BMP-4, 20 ng/mL SCF, and 50 ng/mL VEGF-121 in E8), before centrifugation at 300 × *g* for 3 min. Embryoid bodies were cultured for 4 days, with half medium change after 2 days. Ten to 16 embryoid bodies were plated per well of tissue culture-treated 6-well plates and cultured in 3 mL hematopoetic medium (2 mM GlutaMax, 100 U/mL penicillin, 100 μg/mL streptomycin, 55 μM β-mercaptoethanol, 100 ng/mL M-CSF, and 25 ng/mL IL-3 in X-VIVO 15 [Lonza, LZBE02-060F]). From this point on, 2 mL medium was exchanged every 4–7 days.

PMPs were harvested from suspension during medium exchange and plated in RPMI 1640 at 180,000 cells/cm^2^ in 6-, 12-, or 96-well plates. In the absence of serum, PMPs adhered to uncoated tissue culture-treated plastic within 1 hr, at which point medium was switched to complete microglia medium (10% FBS [Gibco, 16000044 or Sigma, [F2442], 2 mM GlutaMax, 100 U/mL penicillin, 100 μg/mL streptomycin, 100 ng/mL IL-34, and 10 ng/mL GM-CSF in RPMI1640).

Final differentiation of PMPs into microglia occurred over 6–10 days, with full medium change every 2–3 days. All cytokines and growth factors obtained from PeproTech, except for IL-3 (Cell Guidance Systems).

### Statistical Analysis

Statistical analysis was performed in GraphPad Prism 7. Independent genetic backgrounds were considered biological replicates, with wells, treatments, or experiments averaged as technical replicates. *TREM2* genotypes (wild-type, heterozygous, or homozygous for NHD/FTD-like mutations) were grouped for analysis. While genotype n numbers were low, and tests for normality and distribution thus of limited value, this was not considered an impediment to parametric analysis (De Winter, 2013).

## Author Contributions

P.W.B. and F.J.L. conceptualized the study. P.W.B., J.S., and R.S. collected and analyzed most of the experimental data. E.L., H.H., and J.H. collected and provided donor fibroblasts. S.D. analyzed RNA-seq data. P.W.B. and F.J.L. wrote the manuscript. All authors edited and approved the final manuscript.

## Accession Numbers

RNA-seq data are available through NCBI, under accession number GEO: GSE110952.

## Supplemental Information

Document S1. Supplemental Experimental Procedures, Figures S1–S4, and Tables S1–S2

Document S2. Article plus Supplemental Information
