# Supplementary material for: Functional Studies of Missense TREM2 Mutations in Human Stem Cell-Derived Microglia
Source: Stem Cell Reports. 2018 Mar 29;10(4):1294–307. doi: 10.1016/j.stemcr.2018.03.003 (PMC5998752; doi:10.1016/j.stemcr.2018.03.003)
Supplement: Document S1. Supplemental Experimental Procedures, Figures S1–S4, and Tables S1–S2 [file mmc1.pdf]

Stem Cell Reports, Volume *10*

Supplemental Information

Functional Studies of Missense TREM2 Mutations in Human Stem Cell--Derived Microglia

Philip W. Brownjohn, James Smith, Ravi Solanki, Ebba Lohmann, Henry Houlden, John Hardy, Sabine Dietmann, and Frederick J. Livesey

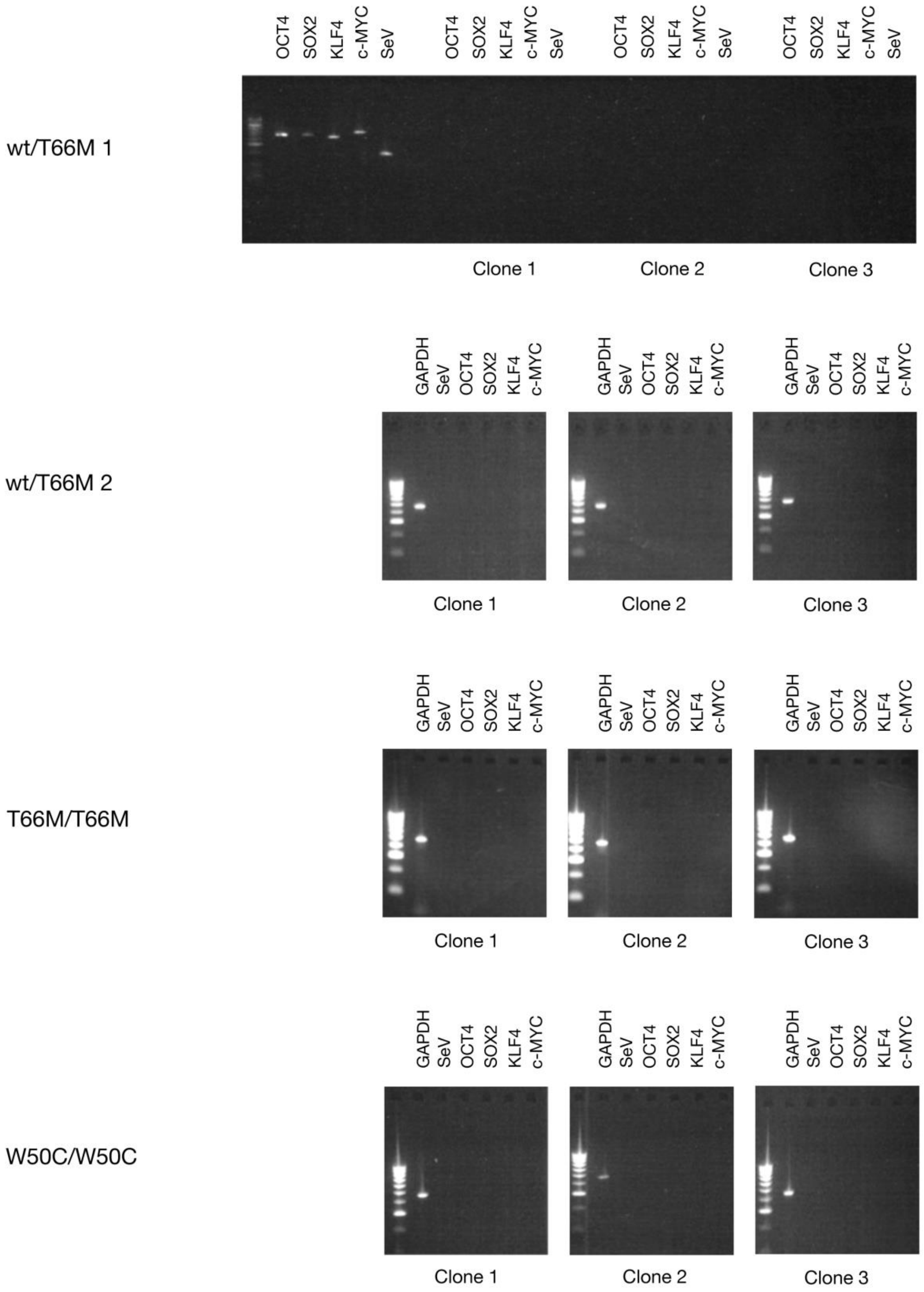
OCT4
SOX2
KLF4
c-MYC
SeV
OCT4
SOX2
KLF4
c-MYC
SeV
OCT4
SOX2
KLF4
c-MYC
SeV
OCT4
SOX2
KLF4
c-MYC
SeV
wt/T66M 1
Clone 1
Clone 2
Clone 3
GAPDH
SeV
OCT4
SOX2
KLF4
c-MYC
wt/T66M 2
Clone 1
Clone 2
Clone 3
T66M/T66M
Clone 1
Clone 2
Clone 3
W50C/W50C
Clone 1
Clone 2
Clone 3

Figure S1. Clearance of Sendai virus from reprogrammed iPSCs. (Relates to *TREM2* mutant lines generated for Figures 4 – 6)

Confirmation of clearance of Sendai reprogramming virus from iPSCs of each genotype and donor, assessed by a lack of Sendai virus RNA (SeV) and absent expression of exogenous transgenes (*OCT4*, *SOX2*, *KLF4*, and *c-MYC*). RT-PCR was carried out for each RNA on three representative clones from each iPSC line. Genotypes and starting fibroblast donor are as labelled.

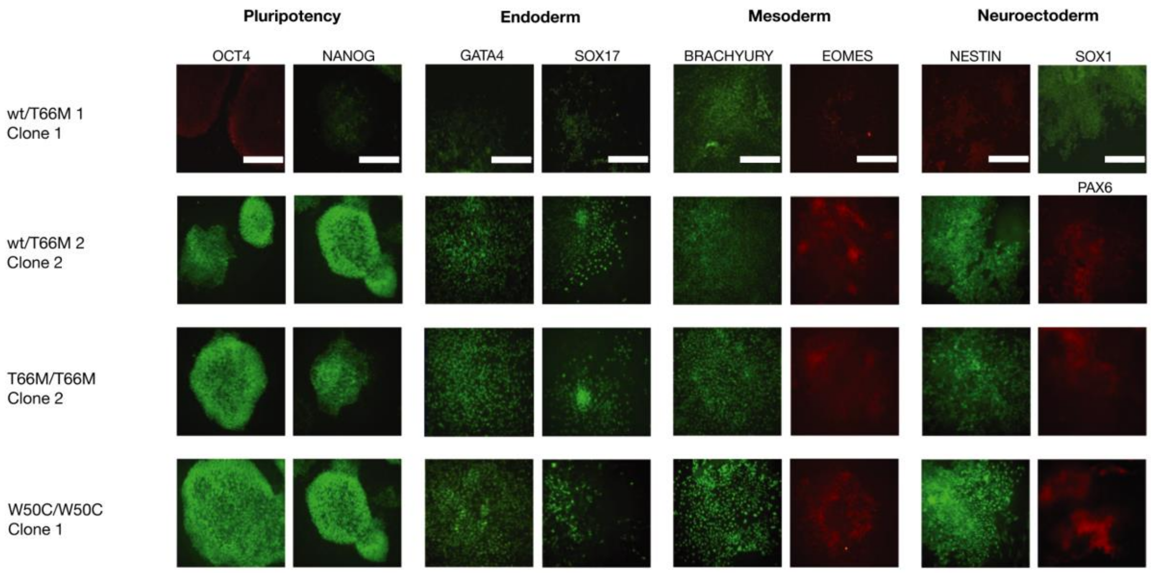

Figure S2. Generation of embryoid bodies and differentiation of a representative iPSC clone from each individual. (Relates to *TREM2* mutant lines generated for Figures 4 – 6)

Expression of pluripotency factors (OCT4, NANOG) was confirmed in starting iPSCs, and expression of markers specific for each of the three major lineages analysed in EBs: endoderm (GATA4, SOX17), mesoderm (BRACHURY, EOMES) and neuroectoderm (NESTIN, SOX1/PAX6). Scale bars represent 400 μm.

wt/T66M 1

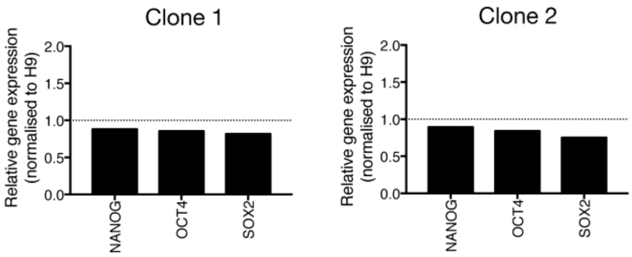

wt/T66M 2

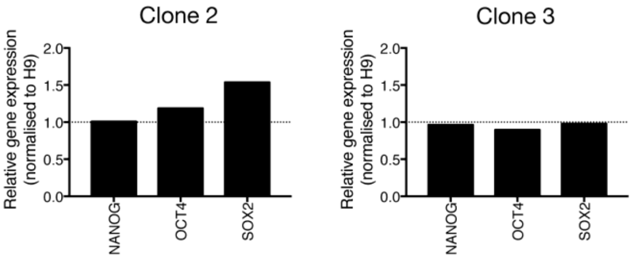

T66M/T66M

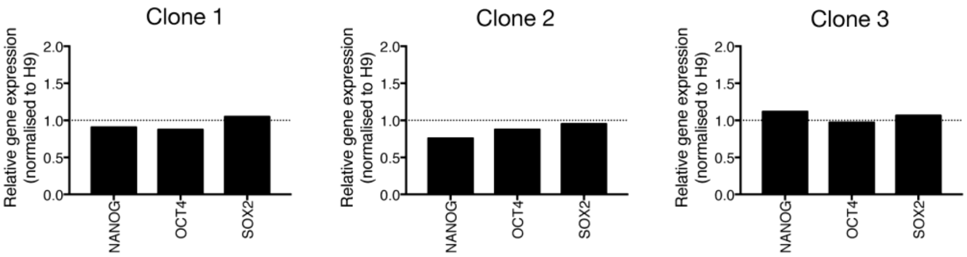

W50C/W50C

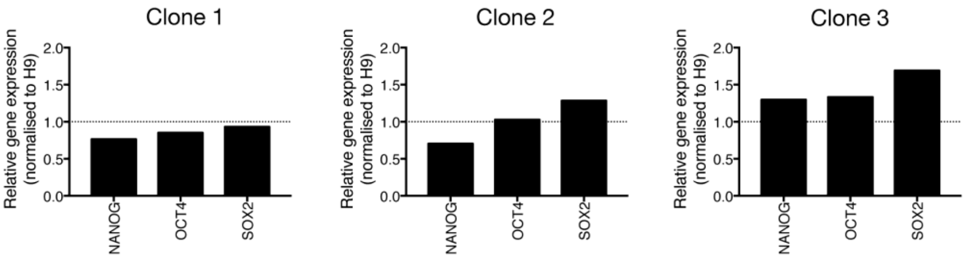

Figure S3. Confirmation of pluripotency of *TREM2* mutant iPSC lines following adaptation to feeder-free culture. (Relates to *TREM2* mutant lines generated for Figures 4 – 6)

Expression of the pluripotency transcripts *NANOG*, *OCT4* and *SOX2* was confirmed in individual clones from each line using a Nanostring nCounter gene expression array. Gene expression levels were normalised to housekeeping genes, and then to expression levels in the pluripotent embryonic stem cell line H9.

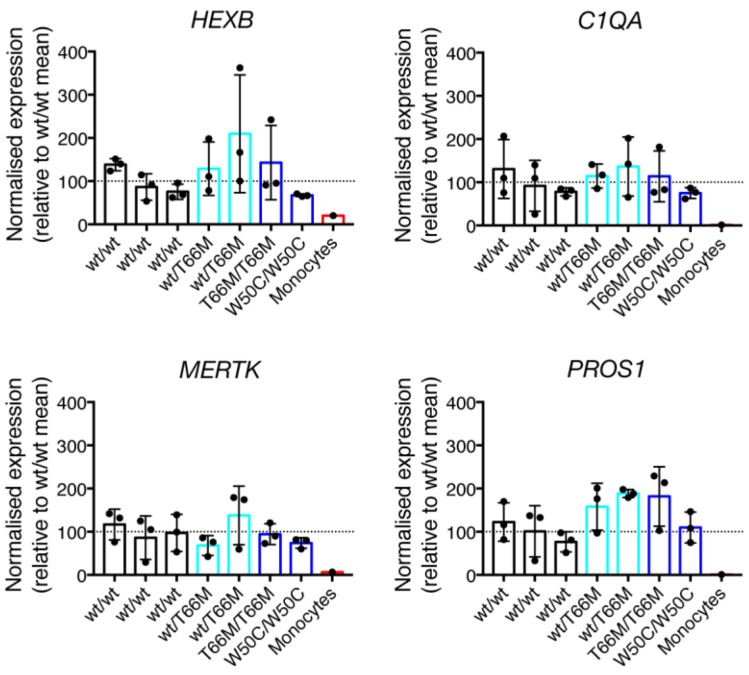

Figure S4. Expression of microglial signature genes in microglia differentiated from wildtype and *TREM2* mutant backgrounds. (Relates to *TREM2* mutant lines generated for Figures 4 – 6)

Microglia differentiated from *TREM2* wildtype (black bars) and heterozygous (cyan bars) and homozygous (dark blue bars) mutant backgrounds express the microglial signature genes *HEXB*, *C1QA*, *MERTK* and *PROS1* as assessed by qPCR. Gene expression levels are shown from three independent differentiations per genetic background, normalised first to the housekeeping genes *ACTB* and *RPS13*, and then to the average expression of all three *TREM2* wildtype backgrounds. As a negative control for these microglia-enriched transcripts, expression levels of each gene are shown for CD14+ peripheral blood monocytes (n = 1). Error bars represent standard deviations.

Hexb	Fscn1	Commd9
Tmem119	Lhfpl2	Adi1
P2ry12	Rcan1	Ndufs3
Lgmn	Cyb5	Mpi
Olfml3	Gpr165	G6pc3
Sgk1	Dnajb4	Dusp7
C1qa	Ctsf	Slc25a4
Plxdc2	CCl2	Tmem258
CCr5	PALDI1	Znf691
Golm1	Cd34	Thrsp
Itgb5	Rps27l	Syngr1
Ltc4s	Gpr155	Tbcb
Bin1	Stau1	Lman1
Lrrc3	Tmem14c	C4orf19
Dynlrb1	COX14	Lap3
Pros1	Upk1b	Ctso
Slc2a5	Itga6	Kiaa0141
Crybb1	FAM212A	Rapgef5
Ecscr	Cxxc5	C9orf69
Cmklr1	Dpm3	Slc39a1
Acp2	Serpinf1	Klhl21
Tanc2	Il1a	Tspan7
Fcgr1A	Tfpi	Stx8
Fcgr1B	Sgce	Arhgap12
Fam102b	Tmem37	Smad1
Snn	Orai1	
Ndufa3	Polr2g	
Ndufc2	Tgfa	
Rtn4rl1	Ubash3b	
Comt	Cryba4	
Etv5	Eif2s1	
Ang	Dctpp1	
LINC00116	Tspan3	
GPR183	P3H2	
Rab3il1	Ndufa7	

Table S1. List of genes upregulated in murine CD45+/TMEM119+ microglia versus CD45+/TMEM119- CNS myeloid cells (Bennett et al., 2016). (Relates to Figure 2)

ACTB_fwd	CACAGAGCCTCGCCTTT
ACTB_rev	GAGCGCGGCGATATCAT
RPS13_fwd	CGAAAGCATCTTGAGAGGAACA
RPS13_rev	TCGAGCCAAACGGTGAATC
HEXB_fwd	GGGAGCATTACGAGGTTTAGAG
HEXB_rev	GGTGGATTCATTGATGGTGAAAG
C1QA_fwd	GGGAAGAAAGGGGAGGCAGG
C1QA_rev	GTTTCCAGAGGGCCCAGGTT
MERTK_fwd	CTTCTCCATGGCCACAGGTT
MERTK_rev	ATACTGAAAAGGTGGGGCGG
PROS1_fwd	AAGAAGCCAGGGAGGTCTTTG
PROS1_rev	ACGTGCAGCAGTGAATAACC

Table S2. Primers used in qPCR. (Relates to Figure S4)

Supplemental Experimental Procedures

Stem cell lines

TREM2 wildtype (wt/wt) cell lines used in this study were AD3 Clone 4 (wt/wt 1), obtained through the IMI StemBANNC consortium, non-demented control Clone 2 (wt/wt 2) (Israel et al., 2012), and the GFP-expressing embryonic stem cell line H9 Cre-LoxP (wt/wt 3) (WiCell).

TREM2 mutation lines were generated from fibroblasts obtained from two donors heterozygous and one patient homozygous for the T66M *TREM2* mutation (2 x wt/T66M; 1 x T66M/T66M), all from the same family, and one patient homozygous for the W50C *TREM2* mutation (W50C/W50C), from skin punch biopsies collected following informed consent and institutional ethics approval. Using non-integrating, four-factor Sendai virus, fibroblasts were reprogrammed through the NIHR Cambridge Biomedical Research Centre (BRC), human Induced Pluripotent Stem Cells core facility. Clones from each genetic background generated for this study were confirmed as free from reprogramming virus by RT-PCR (Figure S1), and pluripotent by differentiation into multiple germ line lineages (Figure S2) and expression of expected pluripotency transcripts (Figure S3). Clones used for primary studies in this paper were: wt/T66M 1 Clone 1; wt/T66M 2 Clone 3; T66M/T66M Clone 1; and W50C/W50C Clone 1.

CytoSNP-850K analysis revealed that, with the exception of wt/T66M donor 2 Clone 3 that was XYY (other clones of this donor untested), all other lines used in this study had expected copy numbers. There was loss of heterozygosity (LoH) on chromosomes 5 (5q13.3 to 5q31.2; 65.1Mb) and 12 (12q13.3 to 12q23.1; 45.5Mb) of wt/T66M donor 2 Clone 3, chromosomes 6 (6p21.2 to 6q22.31; 86.3Mb), 7 (7q11.2 to 7q31.2; 44.9Mb) and 13 (13q14.2 to 13q21.3; 21.4Mb) of all clones derived from patient T66M/T66M, and chromosome 6 (6p22.2 to 6p12.3; 21.4Mb) of all clones derived from patient W50C/W50C. Regions of LoH in patient T66M/T66M, at least in the *TREM2* containing region of Chromosome 6, were previously reported (Guerreiro et al., 2013), and it is hypothesised that this phenomenon precipitated homozygosity of the T66M mutation in this patient. It is likely that this phenomenon also unmasked the recessive W50C mutation in patient W50C/W50C, as the LoH region in this case also encompasses the *TREM2* locus.

Expected *TREM2* mutations were confirmed as present in *TREM2* mutant lines and absent in *TREM2* wildtype lines by Sanger sequencing of exon two of the *TREM2* gene.

Cortical organoid cell culture

Cortical organoids were generated as previously described (Qian et al., 2016) with minor modifications. Briefly, human PSC colonies were dissociated with dispase and cultured overnight in a 10 mL conical tube with Essential 8 medium (day 0). On day 1, colonies were transitioned to ultra-low attachment 6-well plates (Corning) and cultured in Essential 6 medium (Invitrogen) with 2 μM Dorsomorphin (Tocris) and 2 μM A83-01 (Sigma). Media was replaced daily. On day 5-6, half the media was replaced with induction media composed of DMEM:F12 with Glutamax (Invitrogen), 1xN2 Supplement (Invitrogen), 10 μg/mL heparin sulfate (Stem Cell Technologies), 100 U/mL penicillin/streptomycin (Invitrogen), 100 μM Non-essential amino acids (Invitrogen), 4 ng/mL WNT-3A (R&D Systems), 1 μM CHIR99021 (Sigma), and 1 μM SB-431542 (Tocris). On day 7, organoids were embedded in a 2:1 mix of Matrigel (BD Biosciences) and induction media. Induction media was changed daily. On day 14, organoids were mechanically dissociated and cultured in the SpinΩ bioreactor as previously described (Qian et al., 2016).

For microglial infiltration studies, on day 102, cortical organoids were transferred individually to single wells of ultra-low attachment 96-well plates (Corning). Microglia were passaged with Accutase, and resuspended in cortical organoid maturation media (Qian et al., 2016) supplemented with 100 ng/mL IL-34 and 10 ng/mL GM-CSF. 200,000 microglia were added to each organoid. Maturation media was changed every 2 days after the addition of microglia, with IL-34 and GM-CSF removed after the first media change.

Immunofluorescence

After washing in PBS, cell cultures were fixed in 4% (w/vol) paraformaldehyde and blocked with 5% normal donkey serum in 0.3% (vol/vol) Triton-X in TBS before immunofluorescent staining. Unconjugated primary antibodies were directed towards Iba1 (ab5076, Abcam) and TREM2 N-terminal (AF1828, R & D Systems), and PE-conjugated primary antibody was directed towards CD45 (21810454S, Immunotools). Secondary antibodies were Alexa Fluor-conjugated (Thermo Fisher Scientific). For non-permeabilised TREM2 staining, Triton-X was omitted from all blocking and washing steps (Kleinberger et al., 2014).

Cortical organoids and cortical organoid/microglia co-cultures were fixed in 4% (w/vol) paraformaldehyde for 30 minutes at room temperature and cryoprotected with 30% sucrose (w/vol) in PBS overnight. Organoids were embedded in OCT and 12 µm sections generated. Sections were blocked in 5% normal donkey serum in TBS prior to immunofluorescent staining. Primary antibodies were directed to TBR1 (ab31940, Abcam) and SATB2 (ab51502, Abcam), and secondary antibodies were Alexa Fluor-conjugated (Thermo Fisher Scientific).

Imaging of immunocytochemistry and immunohistochemistry was performed on an inverted confocal microscope (Leica TCS SP8 X white light laser) and an Opera Phenix high content imaging system (PerkinElmer), and images analysed with Volocity (PerkinElmer), or Harmony (PerkinElmer). After transfer to 96 well imaging plates (Ibidi), cortical organoid/microglia co-cultures were imaged live with a Marianas inverted 2-photon microscope (3i).

Microglial differentiation efficiency, as determined by labeling with Iba1, CD45 and TREM2, was quantified using the Opera Phenix. Cells were counted as label positive when mean fluorescent intensity exceeded background levels, which was determined by omission of primary antibody (for unconjugated primary antibodies Iba1 and TREM2) or non-specific binding by IgG control (for conjugated primary antibody CD45). Iba1 and CD45 labeling was quantified in microglia differentiated from *TREM2* wildtype and *TREM2* heterozygous and homozygous mutant lines, whereas TREM2 labeling was quantified in *TREM2* wildtype and heterozygous mutants only.

Transcriptome analysis by RNA-Seq

To obtain PMP and microglia RNA samples, three independent differentiations of two genetic backgrounds (n = 5 PMP, n = 6 microglia) were performed. After two PBS washes, RNA was extracted using the RNeasy micro kit (Qiagen) as per manufacturer's instructions, with RIN confirmed as greater than 8.5 in all samples. cDNA libraries were prepared with 250ng of input RNA using a TruSeq Stranded Total RNA kit (RS-122-2201, Illumina), and subjected to 50 bp single end sequencing. RNA-seq reads were quality-trimmed using *Trim Galore!,* and mapped to the human reference genome (GRCh37/hg38) using *TopHat2* with the parameters "--max-multihits 1 --read-mismatch 2 --b2-sensitive". ENSEMBL (release 86) gene models were used to guide alignments with the "–GTF" option. Read counts for genes were obtained using *featureCounts* with the parameters "-p –s 1 –O --minOverlap 10 –B –C". Read counts were normalized, and the statistical significance of differential expression was assessed using the R *Bioconductor DESeq2* package. Gene counts - normalized by *DESeq2* size factors - were subsequently normalized by their effective transcript length/1000, and log2-transformed. Effective transcript lengths were obtained with *featureCounts.* Principal Component Analysis (PCA) was performed using singular value decomposition on scaled expression values with the R *princomp()* function. For FPKM counts, independent differentiations of stem-cell derived microglia were averaged as technical replicates for each genetic background.

Reported gene expression datasets were downloaded from Gene Expression Omnibus (www.ncbi.nlm.nih.gov/geo) under accession numbers GSE73721 (Zhang et al., 2016) (*ex vivo* brain CD45+ microglia/macrophages), GSE55536 (Zhang et al., 2015) (monocyte-derived macrophages), and GSE89189 (Abud et al., 2017) (CD14+/CD16- monocytes, dendritic cells, alternative iPSC-derived microglia, *in vitro* microglia and fetal microglia); or from dbGaP (https://www.ncbi.nlm.nih.gov/gap) under accession number phs001373.v1.p1 (Gosselin et al., 2017) (*ex vivo* and *in vitro* microglia), and processed in the same way. For samples from (Gosselin et al., 2017), only samples from individuals in which matched *ex vivo* and 7 or 10 day *in vitro* cultured (DMEM/F12 + FBS + IL-34) samples were available were used for comparisons.

Gene expression analysis (Nanostring nCounter and RT-PCR)

Expression of the pluripotency genes *NANOG*, *OCT4* and *SOX2* was confirmed in all iPSC lines on the Nanostring nCounter platform using the human stem cell panel of genes, after initial normalisation to housekeeping genes and then expression levels of each transcript in the pluripotent embryonic stem cell line H9 (WiCell).

Semi-quantitative RT-PCR was performed on total RNA from three independent microglial differentiations of each stem cell line used for primary experiments in this study, and CD14+ peripheral blood monocytes (SER-CD14-F, Zenbio). After PBS wash, cells were lysed in RNAlater buffer, and total RNA extracted with the RNeasy mini kit (Qiagen). cDNA was reverse transcribed from RNA with a high-capacity cDNA reverse transcription kit (4368814, Applied Biosystems), using a 1:1 mix of random primers and oligo(dT). qPCR was performed on the StepOnePlus real time PCR system using SYBR Green JumpStart Taq Readymix (S4438, Sigma). Genes of interest were normalised to the geomean of the housekeeping genes *ACTB* and *RPS13*. Primer sequences are supplied in Table S2.

Protein analysis by Immunoblotting

After PBS wash, cells were lysed in 4x LDS sample buffer containing 2 mM DTT. After boiling, cell lysates were subjected to SDS-PAGE and immunoblotting. Primary antibodies used were directed towards calnexin (ADI-SPA-860, Enzo Life Sciences), TREM2 N-terminal (AF1828, R & D Systems) and TREM2 C-terminal (91068, Cell Signalling Technology). Immunoblot detection was performed with Licor secondary antibodies, and analysis performed using the Odyssey Infrared Imaging System.

Lipopolysaccharide challenge

Lipopolysaccharide (LPS) from E. coli (O55:B5, Sigma) was resuspended in PBS before further dilution in microglia media for use in assays. Microglia were treated with LPS-containing microglia media for 6 or 24 hours, with or without co-administration with IFNγ (20 ng/mL), before extracellular media was collected, spun at 800 X G for 3 min to remove contaminating cells, and resultant supernatant stored at -20°C. Microglial supernatant was analysed with multiplexed ELISA (Meso Scale Diagnostics) allowing parallel assessment of secreted IL-1β, TNFα, and IL-6 protein levels from the same sample. For LPS experiments comparing *TREM2* genotypes, released cytokine levels were normalised to cell number measured at baseline on the day of treatment using the Opera Phenix imaging system (PerkinElmer). All treatments were performed in duplicate or triplicate wells.

Phagocytosis and internalisation assays

To measure phagocytosis of bacterial particles, live imaging was performed on microglia exposed to 50 μg/mL pHrodo red conjugated *E. coli* bioparticles (P35361, Invitrogen). To assess the potentially confounding effect of serum on phagocytosis, bioparticles were presented either in standard serum-containing microglia media, or serum-absent microglia media following overnight serum starvation (18 hours exposure to 1% FBS-containing microglia media). As a positive control for reduced phagocytosis, microglia were pre-treated (for 45 min) and then co-treated with 10 μM of the actin polymerisation inhibitor cytochalasin D. As pHrodo labelled dyes are pH sensitive, and fluoresce preferentially in acidic environments such as endosomes and lysosomes, live imaging could be performed in culture media over two or three hours with a high temporal resolution (every 6 min) in order to assess phagocytosis kinetics.

To measure uptake of acetylated low-density lipoprotein (AcLDL), microglia were treated with 5 μg/mL DiI-complexed human AcLDL (L3484, Invitrogen) in microglia media. Thirty minutes or three hours after exposure, AcLDL containing media was removed, and replaced with serum-free RPMI, containing 0.02% trypan blue in order to quench extracellular fluorescence, before immediate imaging.

Live imaging of pHrodo bioparticle uptake and fixed imaging of DiI-complexed AcLDL was performed on the Opera Phenix imaging platform (PerkinElmer), with uptake analysis of both substrates performed with custom parameters within Harmony (PerkinElmer). For pHrodo bioparticle uptake, area of pHrodo signal in the fluorescent channel was measured and calculated as a proportion of total cell area measured in the digital phase

contrast (DPC) channel, while for AcLDL uptake, average intensity across each well of mean DiI signal per cell was calculated.

SUPPLEMENTARY REFERENCES

Abud, E.M., Ramirez, R.N., Martinez, E.S., Healy, L.M., Nguyen, C.H.H., Newman, S.A., Yeromin, A.V., Scarfone, V.M., Marsh, S.E., Fimbres, C., *et al.* (2017). iPSC-Derived Human Microglia-like Cells to Study Neurological Diseases. Neuron *94*, 278-293 e279.

Bennett, M.L., Bennett, F.C., Liddelow, S.A., Ajami, B., Zamanian, J.L., Fernhoff, N.B., Mulinyawe, S.B., Bohlen, C.J., Adil, A., Tucker, A., *et al.* (2016). New tools for studying microglia in the mouse and human CNS. Proceedings of the National Academy of Sciences of the United States of America *113*, E1738-1746.

Gosselin, D., Skola, D., Coufal, N.G., Holtman, I.R., Schlachetzki, J.C.M., Sajti, E., Jaeger, B.N., O'Connor, C., Fitzpatrick, C., Pasillas, M.P., *et al.* (2017). An environment-dependent transcriptional network specifies human microglia identity. Science *356*.

Guerreiro, R.J., Lohmann, E., Bras, J.M., Gibbs, J.R., Rohrer, J.D., Gurunlian, N., Dursun, B., Bilgic, B., Hanagasi, H., Gurvit, H., *et al.* (2013). Using exome sequencing to reveal mutations in TREM2 presenting as a frontotemporal dementia-like syndrome without bone involvement. JAMA Neurol *70*, 78-84.

Israel, M.A., Yuan, S.H., Bardy, C., Reyna, S.M., Mu, Y., Herrera, C., Hefferan, M.P., Van Gorp, S., Nazor, K.L., Boscolo, F.S., *et al.* (2012). Probing sporadic and familial Alzheimer's disease using induced pluripotent stem cells. Nature *482*, 216-220.

Kleinberger, G., Yamanishi, Y., Suarez-Calvet, M., Czirr, E., Lohmann, E., Cuyvers, E., Struyfs, H., Pettkus, N., Wenninger-Weinzierl, A., Mazaheri, F., *et al.* (2014). TREM2 mutations implicated in neurodegeneration impair cell surface transport and phagocytosis. Science translational medicine *6*, 243ra286.

Qian, X., Nguyen, H.N., Song, M.M., Hadiono, C., Ogden, S.C., Hammack, C., Yao, B., Hamersky, G.R., Jacob, F., Zhong, C., *et al.* (2016). Brain-Region-Specific Organoids Using Mini-bioreactors for Modeling ZIKV Exposure. Cell *165*, 1238-1254.

Zhang, H., Xue, C., Shah, R., Bermingham, K., Hinkle, C.C., Li, W., Rodrigues, A., Tabita-Martinez, J., Millar, J.S., Cuchel, M., *et al.* (2015). Functional analysis and transcriptomic profiling of iPSC-derived macrophages and their application in modeling Mendelian disease. Circ Res *117*, 17-28.

Zhang, Y., Sloan, S.A., Clarke, L.E., Caneda, C., Plaza, C.A., Blumenthal, P.D., Vogel, H., Steinberg, G.K., Edwards, M.S., Li, G., *et al.* (2016). Purification and Characterization of Progenitor and Mature Human Astrocytes Reveals Transcriptional and Functional Differences with Mouse. Neuron *89*, 37-53.
