# Supplementary material for: Functional Studies of Missense TREM2 Mutations in Human Stem Cell-Derived Microglia
Source: Stem Cell Reports. 2018 Mar 29;10(4):1294–307. doi: 10.1016/j.stemcr.2018.03.003 (PMC5998752; doi:10.1016/j.stemcr.2018.03.003)
Supplement: Document S2. Article plus Supplemental Information [file mmc2.pdf]

Stem Cell Reports
Article

OPEN ACCESS

Functional Studies of Missense TREM2 Mutations in Human Stem Cell-Derived Microglia

Philip W. Brownjohn,[1] James Smith,[1] Ravi Solanki,[1] Ebba Lohmann,[2,3] Henry Houlden,[4] John Hardy,[4] Sabine Dietmann,[5] and Frederick J. Livesey[1,*]
[1]The Gurdon Institute, ARUK Stem Cell Research Centre and Department of Biochemistry, University of Cambridge, Cambridge CB2 1QN, UK
[2]Department of Neurodegenerative Diseases, Hertie Institute for Clinical Brain Research, University of Tübingen, Tübingen 72076, Germany
[3]DZNE, German Center for Neurodegenerative Diseases, Tübingen 72076, Germany
[4]Department of Molecular Neuroscience, UCL Institute of Neurology, Queen Square, London WC1N 3BG, UK
[5]Wellcome Trust Medical Research Council Stem Cell Institute, University of Cambridge, Tennis Court Road, Cambridge CB2 1QR, UK
*Correspondence: rick@gurdon.cam.ac.uk
https://doi.org/10.1016/j.stemcr.2018.03.003

SUMMARY

The derivation of microglia from human stem cells provides systems for understanding microglial biology and enables functional studies of disease-causing mutations. We describe a robust method for the derivation of human microglia from stem cells, which are phenotypically and functionally comparable with primary microglia. We used stem cell-derived microglia to study the consequences of missense mutations in the microglial-expressed protein triggering receptor expressed on myeloid cells 2 (TREM2), which are causal for frontotemporal dementia-like syndrome and Nasu-Hakola disease. We find that mutant TREM2 accumulates in its immature form, does not undergo typical proteolysis, and is not trafficked to the plasma membrane. However, in the absence of plasma membrane TREM2, microglia differentiate normally, respond to stimulation with lipopolysaccharide, and are phagocytically competent. These data indicate that dementia-associated *TREM2* mutations have subtle effects on microglia biology, consistent with the adult onset of disease in individuals with these mutations.

INTRODUCTION

Microglia are brain-resident immune cells that perform key functions during nervous system development and homeostasis. After colonization and maturation in the developing CNS (Ginhoux et al., 2010; Ransohoff and Cardona, 2010), microglia shape synaptic connections between neurons through synapse pruning and provision of trophic support (Parkhurst et al., 2013; Schafer et al., 2012). As dynamic surveyors of the brain, microglia respond to damage- and pathogen-associated signals to maintain homeostasis (Koizumi et al., 2007; Nimmerjahn et al., 2005). In addition to developmental and homeostatic functions, it is now well established that microglia and associated neuroinflammation play roles in the progression of a number of neurodegenerative conditions (Perry et al., 2010; Ransohoff, 2016), with variants or mutations in microglia-expressed genes linked directly to disease in some cases (Malik et al., 2015; Paloneva et al., 2002; Sims et al., 2017). While microglial function and dysfunction are involved in many neurodegenerative conditions, their precise contributions to disease pathogenesis and progression are not well understood.

Avenues for the study of microglial biology in disease have primarily been limited to animal models and immortalized cell lines, both of which carry limitations in their ability to approximate primary human microglia. As more is understood about the developmental origin and unique identity of microglia, recent studies have attempted to circumvent this issue by deriving microglia from human induced pluripotent stem cells (iPSCs) in order to study human and cell-type-specific biology and disease (Abud et al., 2017; Douvaras et al., 2017; Haenseler et al., 2017; Muffat et al., 2016; Pandya et al., 2017; Takata et al., 2017). Here we describe and characterize a robust method for the derivation of microglia from human stem cells, which we then used to investigate the expressional and functional consequences of mutations in the microglia-expressed triggering receptor expressed on myeloid cells 2 (TREM2).

TREM2 is a transmembrane receptor expressed on cells of myeloid lineage, including osteoclasts and tissue-specific macrophages such as microglia (Colonna and Wang, 2016). Homozygous mutations in TREM2 or its intracellular signaling partner DAP12 are causal for Nasu-Hakola disease (NHD), which is associated with bone cysts and an early-onset dementia (Paloneva et al., 2000, 2002), while a frontotemporal dementia (FTD)-like syndrome without bone dysfunction has also been described in patients carrying certain *TREM2* mutations (Chouery et al., 2008; Giraldo et al., 2013; Guerreiro et al., 2013b). The recent discovery that heterozygous coding variants in *TREM2* confer an increased risk of Alzheimer's disease (AD) (Guerreiro et al., 2013a; Jin et al., 2014; Jonsson et al., 2013) has reignited interest in understanding the role of this receptor in microglial function.

While the endogenous ligand has not been confirmed, *in vitro* studies have demonstrated binding of TREM2 to

This is an open access article under the CC BY license (http://creativecommons.org/licenses/by/4.0/).

lipoprotein, apolipoprotein, and pathogen- and damage-associated ligands (Atagi et al., 2015; Bailey et al., 2015; Daws et al., 2003; Yeh et al., 2016). FTD-like and NHD mutations in *TREM2* are described as loss-of-function mutations, as they result in reduced cell surface expression and ligand binding (Kleinberger et al., 2014; Kober et al., 2016; Park et al., 2015), while AD-associated variants are thought to reduce the affinity of TREM2 for its ligands (Kober et al., 2016; Yeh et al., 2016). Extensive studies have ascribed a number of functions to TREM2, including regulation of phagocytosis (Hsieh et al., 2009; Kleinberger et al., 2014; Takahashi et al., 2005), cytokine release (Hamerman et al., 2006; Turnbull et al., 2006), chemotaxis (Mazaheri et al., 2017), and cell survival (Wang et al., 2015). While murine models of neurodegenerative disease indicate that loss or dysfunction of TREM2 signaling impacts upon microglial function and disease progression (Ulrich et al., 2014; Yuan et al., 2016), the precise role of TREM2 in microglial biology and the consequences of its dysregulation in neurodegenerative disease pathogenesis remain to be determined. Therefore, we used our method for generating human microglia to study the expression, cellular localization, and function of TREM2 in microglia differentiated from iPSCs derived from individuals carrying *TREM2* mutations causal for FTD-like syndrome and NHD.

RESULTS

Human Stem Cell-Derived Microglia Phenotypically Resemble Primary Microglia

Microglia differ from other adult tissue-resident macrophages in two key ways; firstly, their yolk-sac-derived progenitors arise early in development from a program of primitive hematopoiesis rather than the later definitive hematopoiesis that replaces many tissue-resident macrophages in the developed adult (Ginhoux et al., 2010, 2013; Kierdorf et al., 2013; Schulz et al., 2012), and secondly their transcriptome, reflective of the brain-specific roles they perform, is distinct from other myeloid cells (Bennett et al., 2016; Butovsky et al., 2014; Hickman et al., 2013).

As a starting point for the differentiation of microglia, we followed an established method for the derivation of primitive macrophage precursors (PMPs) from human pluripotent stem cells (PSCs) (Karlsson et al., 2008; van Wilgenburg et al., 2013). It has recently been shown that these precursors are produced in a Myb-independent manner, in a pathway closely recapitulating primitive hematopoiesis (Buchrieser et al., 2017). Two to three weeks after the initiation of differentiation, PMPs are produced continuously in suspension, and can be harvested for further maturation. The PMP generation phase can continue indefinitely and is particularly efficient: in the longest ongoing differentiation in this study, one million PSCs produced between 23 and 52 million PMPs in seven PSC lines over 80 days, similar to previously reported PMP yields using the same method (Haenseler et al., 2017; van Wilgenburg et al., 2013) and microglia yields using a recently described alternative method (Abud et al., 2017). Using complete RPMI1640 containing a combination of granulocyte macrophage colony-stimulating factor (GM-CSF) and interleukin-34 (IL-34) (Ohgidani et al., 2014), we differentiated PMPs over 6–10 days to produce monocultures that morphologically resemble microglia (Figure 1A). Analysis of the proportion of these cells expressing canonical macrophage/microglia markers indicates that this protocol has a high level of efficiency across genetic backgrounds, producing cells 95.6% ± 3.6% positive for Iba1 (mean ± SD, n = 6), 95.0% ± 3.6% positive for CD45 (mean ± SD, n = 6), and 99.5% ± 0.4% positive for TREM2 (mean ± SD, n = 5) (Figure 1B).

To investigate the transcriptional identity of our stem cell-derived microglia in the context of the wider myeloid family, we used RNA sequencing (RNA-seq) to compare the transcriptome of these microglia with a number of published datasets: primary *ex vivo* CNS CD45+ microglia/macrophages (Zhang et al., 2016) and microglia (Gosselin et al., 2017), primary microglia and fetal microglia cultured *in vitro* for 7–10 days (Abud et al., 2017; Gosselin et al., 2017), myeloid cells of alternate lineages (monocyte-derived macrophages (Zhang et al., 2015), CD14+/CD16− monocytes (Abud et al., 2017) and dendritic cells (Abud et al., 2017)), and iPSC-microglia derived using an alternative method (Abud et al., 2017) (Figure 2). The recently published dataset from Gosselin et al. (2017) is a particularly useful addition to our understanding of microglial identity, as it compares the transcriptomes of freshly isolated *ex vivo* microglia with the same cells cultured *in vitro* for 7–10 days, allowing assessment of how culture environment alters the transcriptome. PMPs and microglia derived from three independent differentiations from two genetic backgrounds were harvested for RNA-seq analysis, in order to assess reproducibility of the differentiation process both within and between genetic backgrounds.

At the whole-transcriptome level, PMPs and microglia generated by the method reported here most closely resemble cultured primary microglia (Figure 2A). Due to a lack of unique surface markers, it has historically been difficult to distinguish microglia from other macrophages and cells of myeloid lineage. It is only recently that a distinct transcriptomic profile of microglia has emerged (Bennett et al., 2016; Butovsky et al., 2014; Hickman et al., 2013). Using a subset of genes from a recent study that were enriched in expression in murine microglia compared with other CNS myeloid cells (Table S1) (Bennett et al., 2016), we again

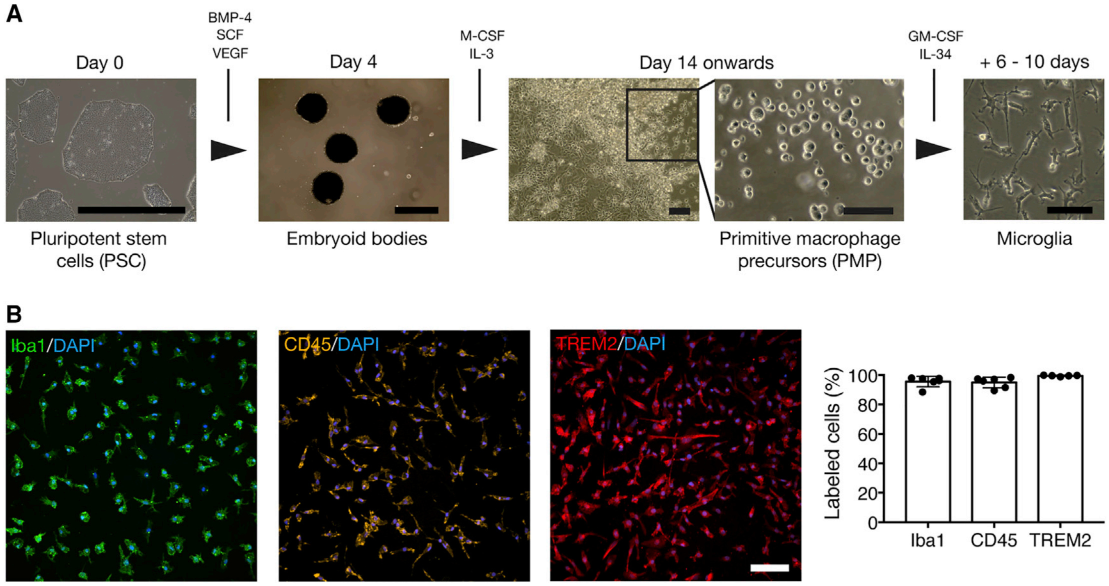

Figure 1. An Efficient Protocol for the Generation of Microglia from Pluripotent Stem Cells

(A) PSCs are differentiated to microglia via embryoid bodies and PMPs. PMPs are produced continuously in culture and are terminally differentiated into microglia when required.

(B) A high proportion of stem cell-derived microglia express the microglial/macrophage markers Iba1, CD45, and TREM2.

Scale bars represent 100 μm, except PSC and embryoid bodies (1 mm). n = 5–6 biological replicates. Error bars represent SDs.

observed that microglia derived from iPSCs using this method cluster most closely with cultured primary microglia, in addition to monocyte-derived macrophages and iPSC-microglia derived using an alternative method (Figure 2B). It is notable that, using this approach, there are three obvious clusters of cell type: *in vitro* cultured microglia/macrophages regardless of origin group together, flanked by a cluster of *ex vivo* microglia and microglia/macrophages and a separate cluster of primary *ex vivo* myeloid cells (dendritic cells and CD14+/CD16− monocytes).

Closer analysis of the expression of microglia "signature" genes (Bennett et al., 2016; Butovsky et al., 2014; Hickman et al., 2013) indicates that stem cell-derived microglia express many of these enriched microglial transcripts at comparable levels with primary *ex vivo* and *in vitro* human microglia (Figure 2C). In sum, these data indicate a close similarity of stem cell-derived microglia with primary microglia at the whole-transcriptome and microglia-specific transcript level.

Functional Characterization of Human Stem Cell-Derived Microglia

Microglia are professional phagocytes, able to clear pathogens and cellular debris. Using live imaging, we found that stem cell-derived microglia efficiently phagocytose bacterial particles, a process attenuated by the actin polymerization inhibitor cytochalasin D (Figure 3A). Microglia express pattern recognition receptors such as Toll-like receptors (TLRs) that mediate responses to pathogenic stimuli. Exposure of microglia to the TLR4 ligand lipopolysaccharide (LPS) alone, or in combination with the immunomodulatory cytokine interferon γ, resulted in an upregulation of the pro-inflammatory cytokines IL-1β ($F_{(2,\ 6)} = 10.26$, $p = 0.0116$), tumor necrosis factor alpha (TNF-α) ($F_{(2,\ 6)} = 20.66$, $p = 0.0020$), and IL-6 ($F_{(2,\ 6)} = 8.848$, $p = 0.0162$) over 24 hr (Figure 3B), confirming that stem cell-derived microglia respond appropriately to pathogenic stimuli.

While maintenance of microglia in monoculture is useful for reductionist studies of defined functions, more complex co-culture models of microglia with neurons have the potential to enable studies of more complex biology, including microglia migration, interactions with neurons, and homing to areas of neuronal injury. To explore the ability of stem cell-derived microglia to invade developing neural tissues and migrate within them, we added microglia to preformed 3D cortical organoids that were cultured in excess of 100 days *in vitro*. Under these conditions, we found that stem cell-derived microglia migrated from the surface deeply into organoids (Figure 3C), and, upon integration, assumed a pronounced ramified morphology, surviving in those environments for at least several weeks in the absence of continued supplementation of the medium with colony-stimulating factors.

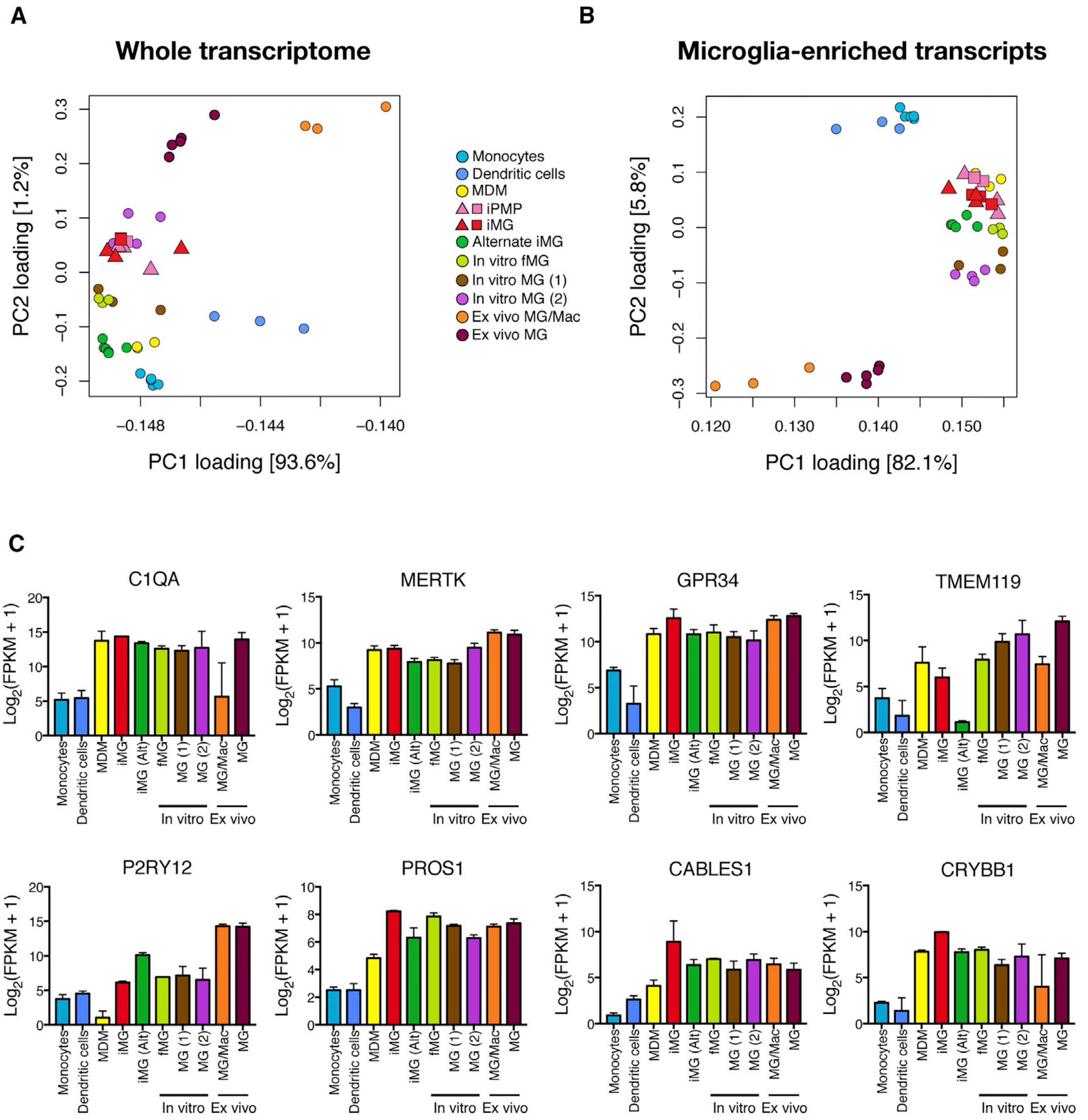

Figure 2. The Transcriptome of Human Stem Cell-Derived Microglia Is Similar to Primary Microglia

(A) At the whole-transcriptome level, iPSC-derived primitive macrophage precursors (iPMPs) and iPSC-derived microglia (iMG) cluster with primary microglia cultured *in vitro* (In vitro MG (2)).

(B) When compared using a subset of genes enriched in murine microglia over other CNS myeloid cells (Bennett et al., 2016), iMG and iPMPs again cluster with primary microglia cultured *in vitro* (In vitro fetal MG [fMG], In vitro MG (1), and In vitro MG (2)), and additionally with an alternative iMG method (Alternate iMG; Abud et al., 2017) and monocyte-derived macrophages (MDM).

(C) FPKM (fragments per kilobase million) counts of "microglia signature" genes indicate comparable levels of expression in iMG compared with *in vitro* and *ex vivo* primary microglia. Monocytes, CD14+/CD16− monocytes (Abud et al., 2017); MDM, monocyte-derived macrophages (Zhang et al., 2015); iPMPs, iPSC-derived primitive macrophage precursors (this study); iMG, iPSC-derived microglia (this study); iMG (alternate), iPSC-microglia derived using an alternative method (Abud et al., 2017); In vitro fMG, *in vitro* fetal microglia (Abud et al., 2017); In vitro MG (1), *in vitro* microglia (Abud et al., 2017); In vitro MG (2), *in vitro* microglia (Gosselin et al., 2017); Ex vivo MG/Mac, primary sorted CNS CD45+ microglia/macrophages (Zhang et al., 2016); Ex vivo MG, primary sorted microglia (Gosselin et al., 2017). For iPMPs and iMG, n = 3 independent differentiations of 2 genetic backgrounds (n = 5 iPMPs, n = 6 iMG).

In (A) and (B), genetic backgrounds of iPMPs and iMG are distinguished by square and triangle symbols. For (C), iMG FPKM values were averaged across differentiations to give values for each genetic background (n = 2). Error bars represent SDs.

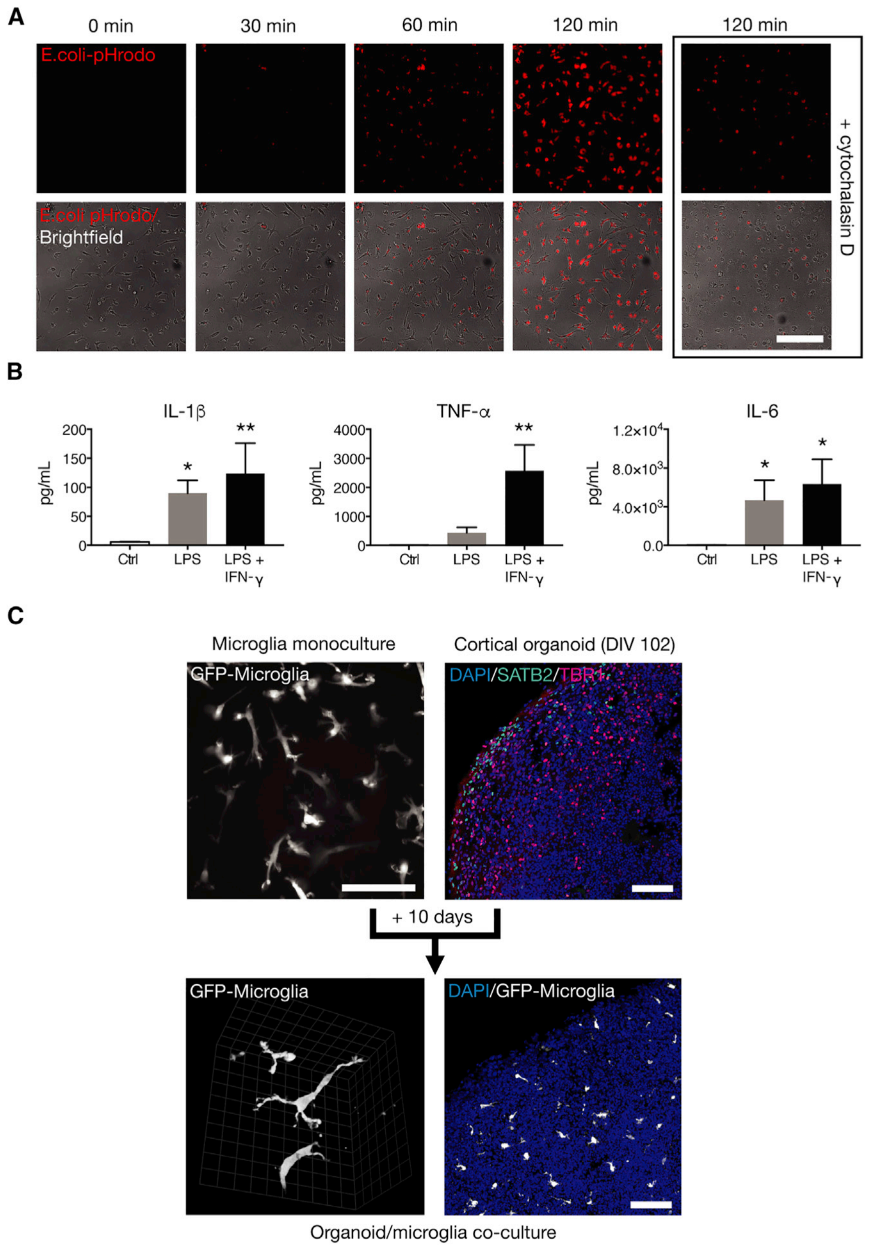

Figure 3. Stem Cell-Derived Microglia Are Functionally Similar to Primary Microglia

(A) Microglia efficiently phagocytose pHrodo-*E. coli*, in a process sensitive to cytochalasin D.

(B) Upon exposure to 100 ng/mL LPS, microglia secrete pro-inflammatory cytokines; an effect augmented by interferon γ (IFN-γ).

(C) Microglia migrate into preformed cortical organoids. Upon migration, microglia tessellate throughout organoids and assume a pronounced ramified morphology, which is demonstrated by live 2-photon imaging of organoid/microglia co-cultures. Scale bars represent 200 μm (A) and 100 μm (C), while scale grid markings at high magnification represent 12.4 μm. In (B), n = 3 biological replicates; * $p < 0.05$, ** $p < 0.01$ treatment versus control; Dunnett's *post hoc* test. Error bars represent SDs.

Expression of Mutant TREM2 in Stem Cell-Derived Microglia

To study the function and dysfunction of TREM2 in microglia, we investigated the most severe form of TREM2 disruption in humans, that of the missense mutations causing FTD-like syndrome and NHD. We obtained fibroblasts from a patient homozygous for the *TREM2* T66M mutation, associated with an FTD-like condition (Guerreiro et al., 2013b) (T66M/T66M), two unaffected family members carrying a single copy of the T66M mutation (wt/T66M), and a patient homozygous for the recently described *TREM2* W50C mutation causal for NHD (Dardiotis et al., 2017) (W50C/W50C), and reprogrammed them to iPSCs. After characterizing iPSC lines (Figures S1–S3), we confirmed their capacity to differentiate into microglia by expression of a number of microglia-enriched genes (Figure S4).

In heterologous cell systems, it has been demonstrated that missense mutations in the *TREM2* gene disrupt intracellular trafficking and protein maturation of TREM2, ultimately reducing functional cell surface expression and ectodomain shedding (Kleinberger et al., 2014; Kober et al., 2016; Park et al., 2015). We sought to determine if cell

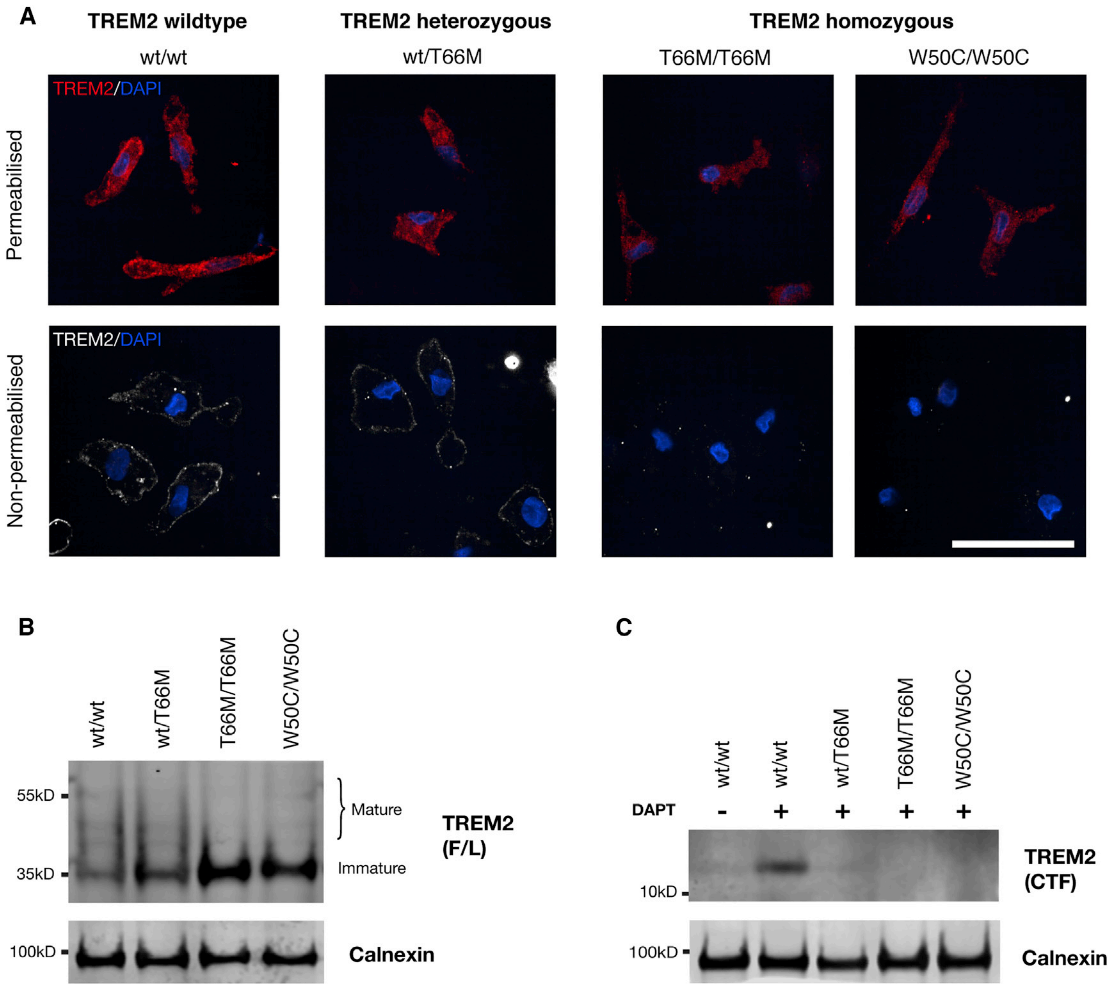

Figure 4. The TREM2 Receptor Is Mislocalized and Aberrantly Processed in *TREM2* Mutant Microglia

(A) Staining for the TREM2 receptor with an N-terminally directed primary antibody reveals expression of TREM2 protein in microglia from *TREM2* wild-type and mutant microglia when cells are permeabilized. Upon omission of permeabilization, TREM2 receptor surface staining is only detected in *TREM2* wild-type and heterozygous mutant microglia, and is absent in microglia derived from homozygous mutant backgrounds.

(B) Probing of whole-cell lysates reveals expression of immature and mature forms of the full-length (F/L) TREM2 receptor in wild-type microglia. Mutations in *TREM2* cause a gene-dosage-dependent accumulation of immature TREM2, and a reduction in mature forms of TREM2.

(C) Probing with a C-terminally directed antibody reveals a weak band corresponding to the CTF of TREM2 in wild-type microglia, indicating efficient turnover of the CTF. Overnight treatment with DAPT (10 μM) results in accumulation of the TREM2-CTF in *TREM2* wild-type microglia, a barely detectable accumulation in heterozygous mutant microglia and no detectable accumulation in homozygous mutant microglia.

Scale bar represents 50 μm.

surface expression, maturation, and proteolysis of TREM2 was impaired in microglia harboring missense mutations in *TREM2* (Figure 4). Using an N-terminal antibody against the extracellular ectodomain of TREM2, we performed immunofluorescence with or without permeabilization of the plasma membrane (Figure 4A). With permeabilization, TREM2 protein was detected in wild-type microglia, as well as microglia from heterozygous (wt/T66M) and homozygous (T66M/T66M and W50C/W50C) *TREM2* mutant backgrounds. In the absence of permeabilization, plasma membrane TREM2 was only detected in wild-type and heterozygous (wt/T66M) *TREM2* backgrounds, indicating a loss of functional receptor surface expression in homozygous *TREM2* mutant microglia.

Western blot analysis of whole-cell lysates indicated that *TREM2* wild-type microglia express both mature and

immature forms of TREM2, but there is a gene dosage-dependent reduction in mature forms and a concomitant increase in immature forms of TREM2 in mutant microglia (Figure 4B), indicating mutation-dependent effects on protein maturation. Full-length TREM2 undergoes regulated intramembrane proteolysis (RIP) by the metalloproteases ADAM17 and ADAM10 (Feuerbach et al., 2017; Kleinberger et al., 2014), which releases a soluble TREM2 fragment and leaves a membrane-bound C-terminal fragment (CTF) that is a substrate for the γ-secretase complex (Wunderlich et al., 2013). To determine the effects of missense mutations on TREM2 RIP, we probed whole-cell lysates with a C-terminally directed TREM2 protein antibody. In the steady state, TREM2-CTF is barely detectable in wild-type microglia, indicating rapid turnover by γ-secretase (Figure 4C). Following treatment with a γ-secretase inhibitor (DAPT), the levels of TREM2 CTF increase in *TREM2* wild-type microglia, but are barely detectable in heterozygous (wt/T66M) and not detectable in homozygous (T66M/T66M and W50C/W50C) *TREM2* mutant microglia, indicating reduced proteolysis of TREM2, most likely due to reduced cell surface expression and shedding of soluble TREM2.

We conclude that the consequences of both *TREM2* missense mutations are complex: there is no detectable TREM2 on the plasma membrane, a reduction in maturation of the TREM2 protein, and reduced generation of the CTF of TREM2. While FTD-like and NHD mutations have been described as loss-of-function mutations, they are not all alike. Nonsense mutations such as Q33X result in a truncated protein, whereas missense mutations such as T66M (and possibly W50C (Dardiotis et al., 2017)) result in aberrant trafficking and either loss of expression or expression of misfolded and non-functional protein on the cell surface (Kleinberger et al., 2014; Kober et al., 2016). In that respect, missense mutations in *TREM2* may not be the equivalent of simple loss-of-function mutations, and may have complex downstream effects on interacting partners and intracellular trafficking machinery.

TREM2 Mutant Microglia Respond Appropriately to Pathogenic Challenge

It has been reported that TREM2 modulates inflammatory responses to pathogenic stimuli (Ito and Hamerman, 2012; Turnbull et al., 2006). We exposed microglia from *TREM2* wild-type and mutant backgrounds to LPS, and measured the release of IL-1β, IL-6, and TNF-α in the extracellular medium after 6 hr (Figure 5A). Two-way ANOVA revealed that, while LPS concentration had a significant effect on the release of IL-1β ($F_{(3, 12)} = 16.94$, $p = 0.0001$), IL-6 ($F_{(3, 12)} = 133.5$, $p = < 0.0001$), and TNF-α ($F_{(3, 12)} = 21.73$, $p = < 0.0001$), there was no significant effect of genotype on cytokine release, and no significant interaction between LPS concentration and genotype. To assess whether clonal differences in reprogrammed iPSC lines might contribute to the observed lack of effect of *TREM2* mutations on response to LPS, we performed further experiments on microglia differentiated from two additional clones of T66M/T66M mutant iPSCs. Again, we found that *TREM2* mutant microglia released similar levels of cytokines to *TREM2* wild-type microglia in response to LPS (Figure 5B). This indicates that *TREM2* mutant microglia have a normal physiological response to pathological stimuli in this system.

TREM2 Mutant Microglia Are Phagocytically Competent

As TREM2 has a proposed role in phagocytosis, we sought to determine if missense mutations in this gene had a significant effect on microglia clearance of bacterial particles. Live imaging over 2 hr with pHrodo-*E. coli* particles revealed that microglia from wild-type *TREM2* backgrounds efficiently phagocytose bioparticles (Figures 6A and 6B). While one-way ANOVA of the 2 hr area under curve (AUC) indicated differences between treatment and genotype groups ($F_{(2, 5)} = 13.42$, $p = 0.0098$), Dunnett's *post hoc* testing revealed that *TREM2* wild-type microglia phagocytosis was significantly inhibited by cytochalasin D ($p = 0.0111$, compared with untreated *TREM2* wild-type microglia); however, microglia differentiated from T66M/T66M and W50C/W50C *TREM2* mutant backgrounds phagocytosed bioparticles as efficiently as microglia from wild-type *TREM2* backgrounds ($p = 0.9519$, compared with untreated *TREM2* wild-type microglia).

Our microglia medium contains serum, which is known to opsonize and enhance phagocytic mechanisms. To investigate whether serum could mask potential deficits in phagocytosis, we repeated *E. coli*-uptake experiments in the absence of serum, following overnight serum starvation (Figures 6C and 6D). In serum-starved conditions, microglia again phagocytosed *E. coil* particles, although notably at a slower rate than in serum-containing conditions. One-way ANOVA of the 3 hr AUC indicated a difference between groups ($F_{(3, 6)} = 18.03$, $p = 0.0021$), which Dunnett's *post hoc* testing revealed was driven by a reduced particle uptake in cytochalasin D-treated *TREM2* wild-type microglia ($p = 0.0042$, compared with untreated *TREM2* wild-type microglia); however, no differences in uptake comparing heterozygous or homozygous *TREM2* mutant microglia with *TREM2* wild-type microglia ($p = 0.3212$ and $p = 0.9361$, respectively, compared with untreated wild-type *TREM2* microglia).

In addition to its proposed role as a phagocytosis receptor, TREM2 has been demonstrated to be an uptake receptor for number of proteins, including acetylated LDL (AcLDL) (Yeh et al., 2016). To assess the uptake of AcLDL in microglia, and the consequences of *TREM2* mutations, microglia from *TREM2* wild-type and homozygous mutant backgrounds were exposed to labeled AcLDL and assessed

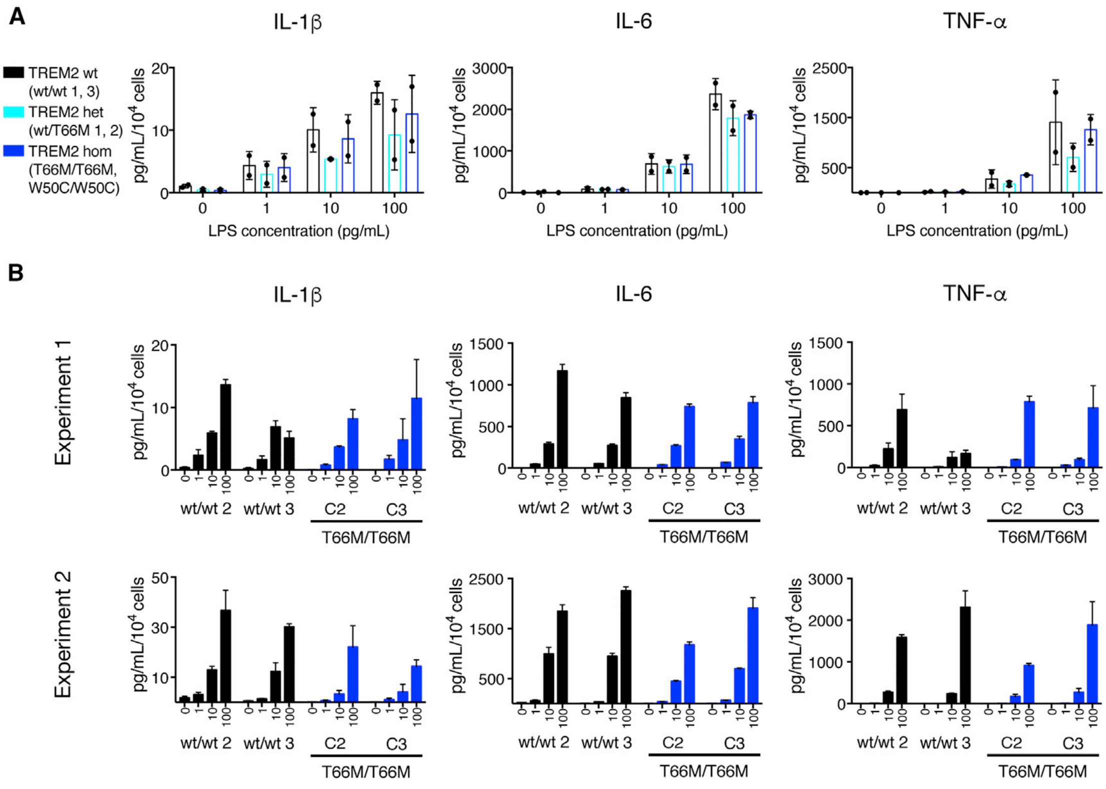

Figure 5. Microglia Harboring TREM2 Mutations Respond Appropriately to LPS Challenge

(A) Upon exposure to LPS, microglia from *TREM2* wild-type and mutant backgrounds release pro-inflammatory cytokines in a dose-dependent manner.

(B) Further experiments in two additional clones from patient T66M/T66M confirm a similar response to LPS challenge across all clones of this genotype.

Results in (A) are an average of all data from two independently performed treatments, n = 2 biological replicates per genotype and error bars represent SDs. Error bars in (B) represent SE of 2–3 technical well replicates, and x-axis values are LPS concentration in pg/mL.

30 min and 3 hr post exposure for internalized AcLDL (Figures 6E–6G). Two-way ANOVA revealed a statistically significant effect of genotype ($F_{(1,\ 6)} = 7.28$, $p = 0.0357$) on the uptake of AcLDL; however, there was no interaction between genotype and time point ($F_{(1,\ 6)} = 0.5492$, $p = 0.4866$), and the effect between genotypes was not significant at either time point alone ($p = 0.3848$ and $p = 0.0995$ at 30 min and 3 hr, respectively), or when compared using the 3 hr uptake AUC ($t_{(3)} = 2.104$, $p = 0.1261$), indicating a minor impairment of AcLDL uptake in *TREM2* homozygous mutant microglia, consistent with the presence of a number of scavenger receptors for AcLDL in addition to TREM2 (Canton et al., 2013).

DISCUSSION

We describe the derivation and characterization of microglia from human stem cells, which transcriptionally and functionally resemble primary microglia. Using these cells as a model for microglial biology, we probed the consequences of *TREM2* mutations using microglia derived from patients carrying missense mutations in *TREM2* causative for the neurodegenerative conditions FTD-like syndrome and NHD. We found that TREM2 is aberrantly processed in microglia from *TREM2* mutant backgrounds, accumulating in its immature form, and, furthermore, is not trafficked appropriately to the cell membrane in homozygous mutant microglia. Unexpectedly, despite dysregulation of TREM2 protein expression, mutant microglia differentiate properly, effectively respond to pathogenic stimuli, and phagocytose appropriately.

Microglia originate from PMPs that invade the developing CNS and mature into microglia (Ginhoux et al., 2010, 2013). To reproduce the developmental origin of microglia, we began with a protocol for the generation of PMPs (Karlsson et al., 2008; van Wilgenburg et al., 2013). These precursors are not dependent on the transcription factor Myb (Buchrieser et al., 2017), and are therefore closer in lineage to yolk-sac-derived precursors of microglia than

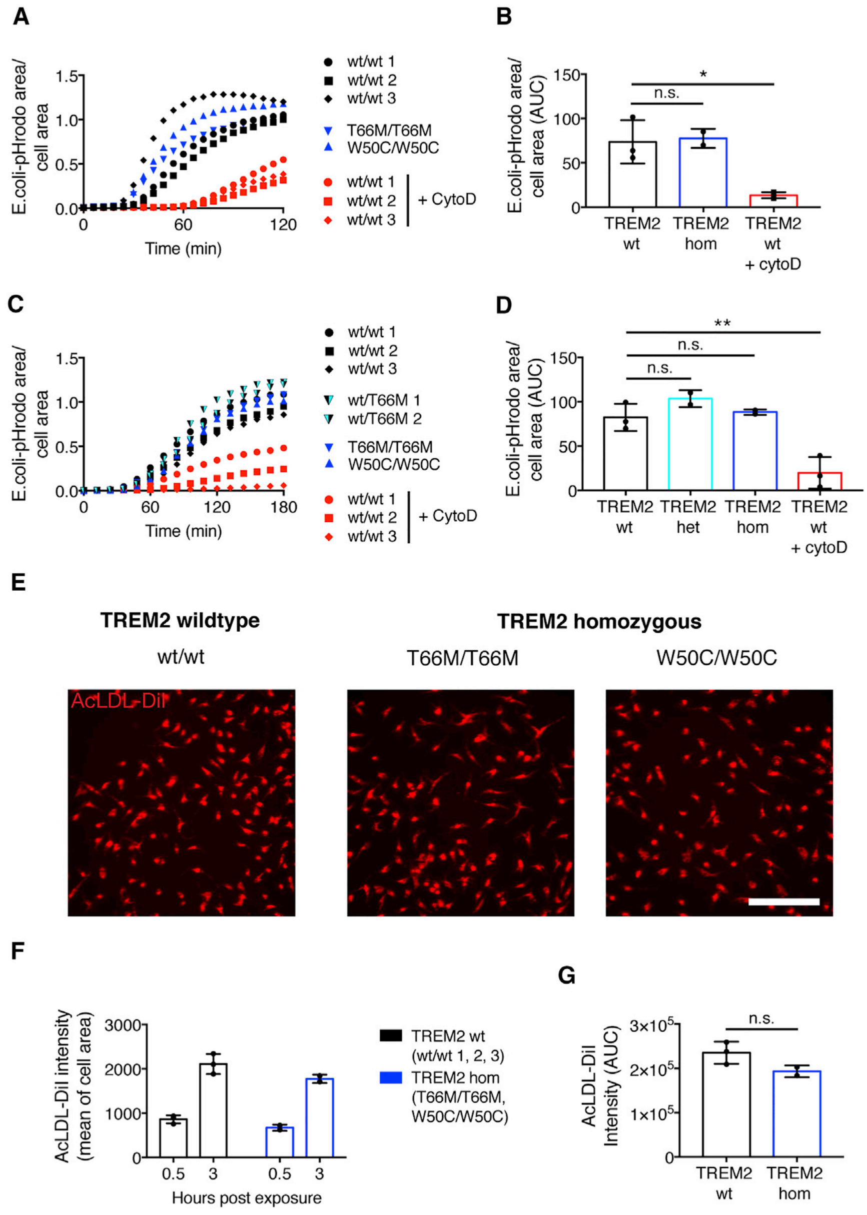

Figure 6. Microglia from *TREM2* Mutant Backgrounds Are Phagocytically Competent

(A and B) Homozygous *TREM2* mutant microglia phagocytose pHrodo-*E. coli* with a similar efficiency and capacity to microglia from a wild-type background. In contrast, cytochalasin D significantly attenuates pHrodo-*E. coli* uptake.

(C and D) Similarly, after serum starvation, *TREM2* mutant microglia phagocytose *E. coli* as efficiently as *TREM2* wild-type microglia.

(E–G) *TREM2* mutant microglia internalize AcLDL almost as efficiently as *TREM2* wild-type microglia.

Scale bar represents 200 μm. All data from two (C, D, and E–G) or three (A and B) independent experiments were averaged to produce values for each genotype. n = 3 (*TREM2* wild-type-untreated; *TREM2* wild-type cytochalasin D-treated) or 2 (*TREM2* heterozygous; *TREM2* homozygous) biological replicates. $*p < 0.05$, $**p < 0.01$ versus untreated *TREM2* wild-type microglia, Dunnett's *post hoc* test. n.s., not significant. Error bars represent SDs.

Myb-dependent precursors of the definitive hematopoiesis pathway (Schulz et al., 2012). Using IL-34, which is produced by neurons in the developing CNS, and is essential for microglial development and survival (Wang et al., 2012), together with GM-CSF, we matured these macrophage precursors into microglia. The entire protocol is rapid and efficient, producing microglia that express a transcriptional and functional signature comparable with primary human microglia within 4 weeks. Over the course of this study, a number of microglia differentiation protocols were reported (Abud et al., 2017; Douvaras et al., 2017; Haenseler et al., 2017; Muffat et al., 2016; Pandya et al., 2017). This method compares favorably with these recent studies, with a simple, sorting-free protocol that yields comparable numbers of microglia over a similar timescale (Abud et al., 2017; Haenseler et al., 2017), and a comparable transcriptome with the microglia described by Abud et al. (2017), which relies on a much more complex differentiation method.

The transcriptional signature associated with microglial identity is rapidly lost upon removal from the neural environment, making isolated culture of microglia challenging (Bohlen et al., 2017; Gosselin et al., 2017). While efforts have been made to understand the environmental cues

driving microglial identity (Bohlen et al., 2017; Butovsky et al., 2014), there are still gaps in our knowledge regarding how medium composition can influence the transcriptome, as further demonstrated in this study, which indicates a clear separation between freshly isolated *ex vivo* and *in vitro* cultured microglia, even between samples from the same individual (Gosselin et al., 2017). Expression of the microglia-enriched gene *TMEM119,* for example, is particularly strongly downregulated in microglia removed from the CNS (Gosselin et al., 2017). The presence of serum in microglial maintenance medium has been reported to induce expression of this gene (Douvaras et al., 2017), however, perhaps explaining why some *TMEM119* expression is observed in our microglia, but not those derived in another study that used defined conditions (Abud et al., 2017). While the use of serum has some drawbacks, including introducing potential variability into differentiation efficiency, we have shown, both transcriptionally and by expression of the macrophage/microglial markers Iba1, CD45, and TREM2, that our differentiation protocol is consistent in efficiency across genetic backgrounds, indicating an acceptable level of variability for studying microglial biology and pathophysiology.

Missense mutations in *TREM2* are thought to affect protein structure, leading to trafficking defects and reduced cell surface expression. We have determined that the FTD-like mutation T66M results in protein mis-processing and reduced surface expression of TREM2 in stem cell-derived microglia, as was previously demonstrated in heterologous cell systems (Kleinberger et al., 2014; Kober et al., 2016; Park et al., 2015). Likewise, the recently identified NHD mutation W50C results in similar mis-regulation, as was predicted by structural modeling (Dardiotis et al., 2017). As TREM2 is postulated to regulate the innate immune system, and is thought to directly modulate phagocytosis and responses to pathogens, we chose to investigate these functions in patient-derived microglia from these *TREM2* mutant backgrounds, and found, surprisingly, that these functions remained intact even in presumed loss-of-function *TREM2* backgrounds.

When TREM2 is overexpressed in non-phagocytic cells, it confers phagocytic activity (Kleinberger et al., 2014; N'Diaye et al., 2009). Likewise, in murine macrophages and microglia, genetic disruption of *TREM2* has been reported to impair the uptake of synthetic material, bacteria, and apoptotic neurons (Hsieh et al., 2009; Kleinberger et al., 2017; Kleinberger et al., 2014; N'Diaye et al., 2009; Takahashi et al., 2005), although this has not always been observed (Wang et al., 2015). It is perhaps surprising that we did not observe impaired phagocytosis in *TREM2* mutant microglia, although the impact of TREM2 disruption on phagocytosis has not previously been demonstrated in human phagocytes expressing physiological levels of TREM2 protein. It is possible that the wide range of phagocytic receptors in microglia (Sierra et al., 2013) acts to compensate for a loss of TREM2, thus masking any potential impairment. Similarly, for the uptake of AcLDL, which we demonstrated is only mildly impaired in microglia derived from *TREM2* mutant backgrounds compared with wild-type backgrounds, there are many receptors that potentially internalize ligands of TREM2, making it difficult to establish a deficit due to loss of function of TREM2 alone. TREM2 has also been demonstrated as a modulator of TLR signaling (Ito and Hamerman, 2012; Turnbull et al., 2006; Wang et al., 2015). While we did not observe differential responses of *TREM2* mutant microglia to the TLR4 ligand LPS, it is known that effects of TREM2 on innate immune responses are variable, and depend upon ligand, cell type, and model system (Ito and Hamerman, 2012; Kang et al., 2018; Turnbull et al., 2006). It is possible that TREM2 has more subtle or context-dependent roles in microglial immune regulation that will require more complex culture conditions to investigate (Keren-Shaul et al., 2017; Krasemann et al., 2017).

In summary, this report details the utility of a simple and effective method for generating microglia from human stem cells. Microglia differentiated from patients carrying missense mutations in *TREM2* that are causal for FTD-like syndrome and NHD accumulate immature TREM2 protein, and do not express functional TREM2 on the cell surface. Despite aberrant TREM2 processing, microglia from mutant *TREM2* backgrounds respond appropriately to challenge with pathogens, releasing similar levels of inflammatory cytokines and phagocytosing as efficiently as *TREM2* wild-type microglia. These results suggest a complex and subtle effect of missense *TREM2* mutations on microglial function that may take some time to manifest in the clinical symptoms, in line with the adult onset of dementia in FTD-like syndrome and NHD.

EXPERIMENTAL PROCEDURES

See Supplemental Information for full details of all methods used.

Microglia Differentiation

Details of PSC lines used in this study can be found in Supplemental Experimental Procedures. *TREM2* mutant lines were generated from fibroblasts derived from skin punch biopsies obtained following informed consent, after approval from the ethics committee of the Istanbul Faculty of Medicine, Istanbul University, or the joint research ethics committee of the National Hospital for Neurology and Neurosurgery and the Institute of Neurology, UCL. Microglia were differentiated from PSCs via embryoid bodies and PMPs (Karlsson et al., 2008; van Wilgenburg et al., 2013). In brief, at least 2 days after last passaging, PSCs (cultured feeder-free in E8) were passaged to single cells with TrypLE Express

(Gibco), and plated at 10,000 per well in 96-well ultra-low attachment plates (Corning) in 100 μL embryoid body medium (10 μM ROCK inhibitor, 50 ng/mL BMP-4, 20 ng/mL SCF, and 50 ng/mL VEGF-121 in E8), before centrifugation at 300 × *g* for 3 min. Embryoid bodies were cultured for 4 days, with half medium change after 2 days. Ten to 16 embryoid bodies were plated per well of tissue culture-treated 6-well plates and cultured in 3 mL hematopoetic medium (2 mM GlutaMax, 100 U/mL penicillin, 100 μg/mL streptomycin, 55 μM β-mercaptoethanol, 100 ng/mL M-CSF, and 25 ng/mL IL-3 in X-VIVO 15 [Lonza, LZBE02-060F]). From this point on, 2 mL medium was exchanged every 4–7 days.

PMPs were harvested from suspension during medium exchange and plated in RPMI 1640 at 180,000 cells/cm^2 in 6-, 12-, or 96-well plates. In the absence of serum, PMPs adhered to uncoated tissue culture-treated plastic within 1 hr, at which point medium was switched to complete microglia medium (10% FBS [Gibco, 16000044 or Sigma, [F2442], 2 mM GlutaMax, 100 U/mL penicillin, 100 μg/mL streptomycin, 100 ng/mL IL-34, and 10 ng/mL GM-CSF in RPMI1640).

Final differentiation of PMPs into microglia occurred over 6–10 days, with full medium change every 2–3 days. All cytokines and growth factors obtained from PeproTech, except for IL-3 (Cell Guidance Systems).

Statistical Analysis

Statistical analysis was performed in GraphPad Prism 7. Independent genetic backgrounds were considered biological replicates, with wells, treatments, or experiments averaged as technical replicates. *TREM2* genotypes (wild-type, heterozygous, or homozygous for NHD/FTD-like mutations) were grouped for analysis. While genotype n numbers were low, and tests for normality and distribution thus of limited value, this was not considered an impediment to parametric analysis (De Winter, 2013).

ACCESSION NUMBERS

RNA-seq data are available through NCBI, under accession number GEO: GSE110952.

SUPPLEMENTAL INFORMATION

Supplemental Information includes Supplemental Experimental Procedures, four figures, and two tables and can be found with this article online at https://doi.org/10.1016/j.stemcr.2018.03.003.

AUTHOR CONTRIBUTIONS

P.W.B. and F.J.L. conceptualized the study. P.W.B., J.S., and R.S. collected and analyzed most of the experimental data. E.L., H.H., and J.H. collected and provided donor fibroblasts. S.D. analyzed RNA-seq data. P.W.B. and F.J.L. wrote the manuscript. All authors edited and approved the final manuscript.

ACKNOWLEDGMENTS

This research was supported by the Alzheimer's Research UK Stem Cell Research Centre, funded by the Alborada Trust (to P.W.B., J.S., and F.J.L.), a Wellcome Trust Senior Investigator Award (to F.J.L.), and Dementias Platform UK. J.S. was funded by the IMI StemBANCC. R.S. is supported by the University of Cambridge MB/PhD program. Research in F.J.L.'s group benefits from core support to the Gurdon Institute from the Wellcome Trust and Cancer Research UK. We thank patients and their family members for donation of fibroblasts, Dr Vickie Stubbs and Ellie Tuck of the Livesey lab for technical advice, and Kay Harnish of the Gurdon Institute for preparation of cDNA libraries.

Received: August 28, 2017
Revised: March 2, 2018
Accepted: March 5, 2018
Published: March 29, 2018

REFERENCES

Abud, E.M., Ramirez, R.N., Martinez, E.S., Healy, L.M., Nguyen, C.H.H., Newman, S.A., Yeromin, A.V., Scarfone, V.M., Marsh, S.E., Fimbres, C., et al. (2017). iPSC-derived human microglia-like cells to study neurological diseases. Neuron *94*, 278–293.e9.

Atagi, Y., Liu, C.C., Painter, M.M., Chen, X.F., Verbeeck, C., Zheng, H., Li, X., Rademakers, R., Kang, S.S., Xu, H., et al. (2015). Apolipoprotein E is a ligand for triggering receptor expressed on myeloid cells 2 (TREM2). J. Biol. Chem. *290*, 26043–26050.

Bailey, C.C., DeVaux, L.B., and Farzan, M. (2015). The triggering receptor expressed on myeloid cells 2 binds apolipoprotein E. J. Biol. Chem. *290*, 26033–26042.

Bennett, M.L., Bennett, F.C., Liddelow, S.A., Ajami, B., Zamanian, J.L., Fernhoff, N.B., Mulinyawe, S.B., Bohlen, C.J., Adil, A., Tucker, A., et al. (2016). New tools for studying microglia in the mouse and human CNS. Proc. Natl. Acad. Sci. USA *113*, E1738–E1746.

Bohlen, C.J., Bennett, F.C., Tucker, A.F., Collins, H.Y., Mulinyawe, S.B., and Barres, B.A. (2017). Diverse requirements for microglial survival, specification, and function revealed by defined-medium cultures. Neuron *94*, 759–773.e8.

Buchrieser, J., James, W., and Moore, M.D. (2017). Human induced pluripotent stem cell-derived macrophages share ontogeny with MYB-independent tissue-resident macrophages. Stem Cell Reports *8*, 334–345.

Butovsky, O., Jedrychowski, M.P., Moore, C.S., Cialic, R., Lanser, A.J., Gabriely, G., Koeglsperger, T., Dake, B., Wu, P.M., Doykan, C.E., et al. (2014). Identification of a unique TGF-beta-dependent molecular and functional signature in microglia. Nat. Neurosci. *17*, 131–143.

Canton, J., Neculai, D., and Grinstein, S. (2013). Scavenger receptors in homeostasis and immunity. Nat. Rev. Immunol. *13*, 621–634.

Chouery, E., Delague, V., Bergougnoux, A., Koussa, S., Serre, J.L., and Megarbane, A. (2008). Mutations in TREM2 lead to pure early-onset dementia without bone cysts. Hum. Mutat. *29*, E194–E204.

Colonna, M., and Wang, Y. (2016). TREM2 variants: new keys to decipher Alzheimer disease pathogenesis. Nat. Rev. Neurosci. *17*, 201–207.

Dardiotis, E., Siokas, V., Pantazi, E., Dardioti, M., Rikos, D., Xiromerisiou, G., Markou, A., Papadimitriou, D., Speletas, M., and

Hadjigeorgiou, G.M. (2017). A novel mutation in TREM2 gene causing Nasu-Hakola disease and review of the literature. Neurobiol. Aging *53*, 194.e13–194.e22.

Daws, M.R., Sullam, P.M., Niemi, E.C., Chen, T.T., Tchao, N.K., and Seaman, W.E. (2003). Pattern recognition by TREM-2: binding of anionic ligands. J. Immunol. *171*, 594–599.

De Winter, J.C. (2013). Using the Student's t-test with extremely small sample sizes. Practical Assessment Research & Evaluation 18. Available online: http://pareonline.net/getvn.asp?v=18&n=10.

Douvaras, P., Sun, B., Wang, M., Kruglikov, I., Lallos, G., Zimmer, M., Terrenoire, C., Zhang, B., Gandy, S., Schadt, E., et al. (2017). Directed differentiation of human pluripotent stem cells to microglia. Stem Cell Reports *8*, 1516–1524.

Feuerbach, D., Schindler, P., Barske, C., Joller, S., Beng-Louka, E., Worringer, K.A., Kommineni, S., Kaykas, A., Ho, D.J., Ye, C., et al. (2017). ADAM17 is the main sheddase for the generation of human triggering receptor expressed in myeloid cells (hTREM2) ectodomain and cleaves TREM2 after Histidine 157. Neurosci. Lett. *660*, 109–114.

Ginhoux, F., Greter, M., Leboeuf, M., Nandi, S., See, P., Gokhan, S., Mehler, M.F., Conway, S.J., Ng, L.G., Stanley, E.R., et al. (2010). Fate mapping analysis reveals that adult microglia derive from primitive macrophages. Science *330*, 841–845.

Ginhoux, F., Lim, S., Hoeffel, G., Low, D., and Huber, T. (2013). Origin and differentiation of microglia. Front. Cell. Neurosci. *7*, 45.

Giraldo, M., Lopera, F., Siniard, A.L., Corneveaux, J.J., Schrauwen, I., Carvajal, J., Munoz, C., Ramirez-Restrepo, M., Gaiteri, C., Myers, A.J., et al. (2013). Variants in triggering receptor expressed on myeloid cells 2 are associated with both behavioral variant frontotemporal lobar degeneration and Alzheimer's disease. Neurobiol. Aging *34*, 2077.e11–2077.e18.

Gosselin, D., Skola, D., Coufal, N.G., Holtman, I.R., Schlachetzki, J.C.M., Sajti, E., Jaeger, B.N., O'Connor, C., Fitzpatrick, C., Pasillas, M.P., et al. (2017). An environment-dependent transcriptional network specifies human microglia identity. Science *356*. https://doi.org/10.1126/science.aal3222.

Guerreiro, R., Wojtas, A., Bras, J., Carrasquillo, M., Rogaeva, E., Majounie, E., Cruchaga, C., Sassi, C., Kauwe, J.S., Younkin, S., et al. (2013a). TREM2 variants in Alzheimer's disease. N. Engl. J. Med. *368*, 117–127.

Guerreiro, R.J., Lohmann, E., Bras, J.M., Gibbs, J.R., Rohrer, J.D., Gurunlian, N., Dursun, B., Bilgic, B., Hanagasi, H., Gurvit, H., et al. (2013b). Using exome sequencing to reveal mutations in TREM2 presenting as a frontotemporal dementia-like syndrome without bone involvement. JAMA Neurol. *70*, 78–84.

Haenseler, W., Sansom, S.N., Buchrieser, J., Newey, S.E., Moore, C.S., Nicholls, F.J., Chintawar, S., Schnell, C., Antel, J.P., Allen, N.D., et al. (2017). A highly efficient human pluripotent stem cell microglia model displays a neuronal-co-culture-specific expression profile and inflammatory response. Stem Cell Reports *8*, 1727–1742.

Hamerman, J.A., Jarjoura, J.R., Humphrey, M.B., Nakamura, M.C., Seaman, W.E., and Lanier, L.L. (2006). Cutting edge: inhibition of TLR and FcR responses in macrophages by triggering receptor expressed on myeloid cells (TREM)-2 and DAP12. J. Immunol. *177*, 2051–2055.

Hickman, S.E., Kingery, N.D., Ohsumi, T.K., Borowsky, M.L., Wang, L.C., Means, T.K., and El Khoury, J. (2013). The microglial sensome revealed by direct RNA sequencing. Nat. Neurosci. *16*, 1896–1905.

Hsieh, C.L., Koike, M., Spusta, S.C., Niemi, E.C., Yenari, M., Nakamura, M.C., and Seaman, W.E. (2009). A role for TREM2 ligands in the phagocytosis of apoptotic neuronal cells by microglia. J. Neurochem. *109*, 1144–1156.

Ito, H., and Hamerman, J.A. (2012). TREM-2, triggering receptor expressed on myeloid cell-2, negatively regulates TLR responses in dendritic cells. Eur. J. Immunol. *42*, 176–185.

Jin, S.C., Benitez, B.A., Karch, C.M., Cooper, B., Skorupa, T., Carrell, D., Norton, J.B., Hsu, S., Harari, O., Cai, Y., et al. (2014). Coding variants in TREM2 increase risk for Alzheimer's disease. Hum. Mol. Genet. *23*, 5838–5846.

Jonsson, T., Stefansson, H., Steinberg, S., Jonsdottir, I., Jonsson, P.V., Snaedal, J., Bjornsson, S., Huttenlocher, J., Levey, A.I., Lah, J.J., et al. (2013). Variant of TREM2 associated with the risk of Alzheimer's disease. N. Engl. J. Med. *368*, 107–116.

Kang, S.S., Kurti, A., Baker, K.E., Liu, C.C., Colonna, M., Ulrich, J.D., Holtzman, D.M., Bu, G., and Fryer, J.D. (2018). Behavioral and transcriptomic analysis of Trem2-null mice: not all knockout mice are created equal. Hum. Mol. Genet. *27*, 211–223.

Karlsson, K.R., Cowley, S., Martinez, F.O., Shaw, M., Minger, S.L., and James, W. (2008). Homogeneous monocytes and macrophages from human embryonic stem cells following coculture-free differentiation in M-CSF and IL-3. Exp. Hematol. *36*, 1167–1175.

Keren-Shaul, H., Spinrad, A., Weiner, A., Matcovitch-Natan, O., Dvir-Szternfeld, R., Ulland, T.K., David, E., Baruch, K., Lara-Astaiso, D., Toth, B., et al. (2017). A unique microglia type associated with restricting development of Alzheimer's disease. Cell *169*, 1276–1290.e17.

Kierdorf, K., Erny, D., Goldmann, T., Sander, V., Schulz, C., Perdiguero, E.G., Wieghofer, P., Heinrich, A., Riemke, P., Holscher, C., et al. (2013). Microglia emerge from erythromyeloid precursors via Pu.1- and Irf8-dependent pathways. Nat. Neurosci. *16*, 273–280.

Kleinberger, G., Brendel, M., Mracsko, E., Wefers, B., Groeneweg, L., Xiang, X., Focke, C., Deussing, M., Suarez-Calvet, M., Mazaheri, F., et al. (2017). The FTD-like syndrome causing TREM2 T66M mutation impairs microglia function, brain perfusion, and glucose metabolism. EMBO J. *36*, 1837–1853.

Kleinberger, G., Yamanishi, Y., Suarez-Calvet, M., Czirr, E., Lohmann, E., Cuyvers, E., Struyfs, H., Pettkus, N., Wenninger-Weinzierl, A., Mazaheri, F., et al. (2014). TREM2 mutations implicated in neurodegeneration impair cell surface transport and phagocytosis. Sci. Transl. Med. *6*, 243ra286.

Kober, D.L., Alexander-Brett, J.M., Karch, C.M., Cruchaga, C., Colonna, M., Holtzman, M.J., and Brett, T.J. (2016). Neurodegenerative disease mutations in TREM2 reveal a functional surface and distinct loss-of-function mechanisms. Elife 5. https://doi.org/10.7554/eLife.20391.

Koizumi, S., Shigemoto-Mogami, Y., Nasu-Tada, K., Shinozaki, Y., Ohsawa, K., Tsuda, M., Joshi, B.V., Jacobson, K.A., Kohsaka, S.,

and Inoue, K. (2007). UDP acting at P2Y6 receptors is a mediator of microglial phagocytosis. Nature *446*, 1091–1095.

Krasemann, S., Madore, C., Cialic, R., Baufeld, C., Calcagno, N., El Fatimy, R., Beckers, L., O'Loughlin, E., Xu, Y., Fanek, Z., et al. (2017). The TREM2-APOE pathway drives the transcriptional phenotype of dysfunctional microglia in neurodegenerative diseases. Immunity *47*, 566–581.e9.

Malik, M., Parikh, I., Vasquez, J.B., Smith, C., Tai, L., Bu, G., LaDu, M.J., Fardo, D.W., Rebeck, G.W., and Estus, S. (2015). Genetics ignite focus on microglial inflammation in Alzheimer's disease. Mol. Neurodegener. *10*, 52.

Mazaheri, F., Snaidero, N., Kleinberger, G., Madore, C., Daria, A., Werner, G., Krasemann, S., Capell, A., Trumbach, D., Wurst, W., et al. (2017). TREM2 deficiency impairs chemotaxis and microglial responses to neuronal injury. EMBO Rep. *18*, 1186–1198.

Muffat, J., Li, Y., Yuan, B., Mitalipova, M., Omer, A., Corcoran, S., Bakiasi, G., Tsai, L.H., Aubourg, P., Ransohoff, R.M., et al. (2016). Efficient derivation of microglia-like cells from human pluripotent stem cells. Nat. Med. *22*, 1358–1367.

N'Diaye, E.N., Branda, C.S., Branda, S.S., Nevarez, L., Colonna, M., Lowell, C., Hamerman, J.A., and Seaman, W.E. (2009). TREM-2 (triggering receptor expressed on myeloid cells 2) is a phagocytic receptor for bacteria. J. Cell Biol. *184*, 215–223.

Nimmerjahn, A., Kirchhoff, F., and Helmchen, F. (2005). Resting microglial cells are highly dynamic surveillants of brain parenchyma in vivo. Science *308*, 1314–1318.

Ohgidani, M., Kato, T.A., Setoyama, D., Sagata, N., Hashimoto, R., Shigenobu, K., Yoshida, T., Hayakawa, K., Shimokawa, N., Miura, D., et al. (2014). Direct induction of ramified microglia-like cells from human monocytes: dynamic microglial dysfunction in Nasu-Hakola disease. Sci. Rep. *4*, 4957.

Paloneva, J., Kestila, M., Wu, J., Salminen, A., Bohling, T., Ruotsalainen, V., Hakola, P., Bakker, A.B., Phillips, J.H., Pekkarinen, P., et al. (2000). Loss-of-function mutations in TYROBP (DAP12) result in a presenile dementia with bone cysts. Nat. Genet. *25*, 357–361.

Paloneva, J., Manninen, T., Christman, G., Hovanes, K., Mandelin, J., Adolfsson, R., Bianchin, M., Bird, T., Miranda, R., Salmaggi, A., et al. (2002). Mutations in two genes encoding different subunits of a receptor signaling complex result in an identical disease phenotype. Am. J. Hum. Genet. *71*, 656–662.

Pandya, H., Shen, M.J., Ichikawa, D.M., Sedlock, A.B., Choi, Y., Johnson, K.R., Kim, G., Brown, M.A., Elkahloun, A.G., Maric, D., et al. (2017). Differentiation of human and murine induced pluripotent stem cells to microglia-like cells. Nat. Neurosci. *20*, 753–759.

Park, J.S., Ji, I.J., An, H.J., Kang, M.J., Kang, S.W., Kim, D.H., and Yoon, S.Y. (2015). Disease-associated mutations of TREM2 alter the processing of N-linked oligosaccharides in the Golgi apparatus. Traffic *16*, 510–518.

Parkhurst, C.N., Yang, G., Ninan, I., Savas, J.N., Yates, J.R., 3rd, Lafaille, J.J., Hempstead, B.L., Littman, D.R., and Gan, W.B. (2013). Microglia promote learning-dependent synapse formation through brain-derived neurotrophic factor. Cell *155*, 1596–1609.

Perry, V.H., Nicoll, J.A., and Holmes, C. (2010). Microglia in neurodegenerative disease. Nat. Rev. Neurol. *6*, 193–201.

Ransohoff, R.M. (2016). How neuroinflammation contributes to neurodegeneration. Science *353*, 777–783.

Ransohoff, R.M., and Cardona, A.E. (2010). The myeloid cells of the central nervous system parenchyma. Nature *468*, 253–262.

Schafer, D.P., Lehrman, E.K., Kautzman, A.G., Koyama, R., Mardinly, A.R., Yamasaki, R., Ransohoff, R.M., Greenberg, M.E., Barres, B.A., and Stevens, B. (2012). Microglia sculpt postnatal neural circuits in an activity and complement-dependent manner. Neuron *74*, 691–705.

Schulz, C., Gomez Perdiguero, E., Chorro, L., Szabo-Rogers, H., Cagnard, N., Kierdorf, K., Prinz, M., Wu, B., Jacobsen, S.E., Pollard, J.W., et al. (2012). A lineage of myeloid cells independent of Myb and hematopoietic stem cells. Science *336*, 86–90.

Sierra, A., Abiega, O., Shahraz, A., and Neumann, H. (2013). Janus-faced microglia: beneficial and detrimental consequences of microglial phagocytosis. Front. Cell. Neurosci. *7*, 6.

Sims, R., van der Lee, S.J., Naj, A.C., Bellenguez, C., Badarinarayan, N., Jakobsdottir, J., Kunkle, B.W., Boland, A., Raybould, R., Bis, J.C., et al. (2017). Rare coding variants in PLCG2, ABI3, and TREM2 implicate microglial-mediated innate immunity in Alzheimer's disease. Nat. Genet. *49*, 1373–1384.

Takahashi, K., Rochford, C.D., and Neumann, H. (2005). Clearance of apoptotic neurons without inflammation by microglial triggering receptor expressed on myeloid cells-2. J. Exp. Med. *201*, 647–657.

Takata, K., Kozaki, T., Lee, C.Z.W., Thion, M.S., Otsuka, M., Lim, S., Utami, K.H., Fidan, K., Park, D.S., Malleret, B., et al. (2017). Induced-pluripotent-stem-cell-derived primitive macrophages provide a platform for modeling tissue-resident macrophage differentiation and function. Immunity *47*, 183–198.e6.

Turnbull, I.R., Gilfillan, S., Cella, M., Aoshi, T., Miller, M., Piccio, L., Hernandez, M., and Colonna, M. (2006). Cutting edge: TREM-2 attenuates macrophage activation. J. Immunol. *177*, 3520–3524.

Ulrich, J.D., Finn, M.B., Wang, Y., Shen, A., Mahan, T.E., Jiang, H., Stewart, F.R., Piccio, L., Colonna, M., and Holtzman, D.M. (2014). Altered microglial response to Abeta plaques in APPPS1-21 mice heterozygous for TREM2. Mol. Neurodegener. *9*, 20.

van Wilgenburg, B., Browne, C., Vowles, J., and Cowley, S.A. (2013). Efficient, long term production of monocyte-derived macrophages from human pluripotent stem cells under partly-defined and fully-defined conditions. PLoS One *8*, e71098.

Wang, Y., Cella, M., Mallinson, K., Ulrich, J.D., Young, K.L., Robinette, M.L., Gilfillan, S., Krishnan, G.M., Sudhakar, S., Zinselmeyer, B.H., et al. (2015). TREM2 lipid sensing sustains the microglial response in an Alzheimer's disease model. Cell *160*, 1061–1071.

Wang, Y., Szretter, K.J., Vermi, W., Gilfillan, S., Rossini, C., Cella, M., Barrow, A.D., Diamond, M.S., and Colonna, M. (2012). IL-34 is a tissue-restricted ligand of CSF1R required for the development of Langerhans cells and microglia. Nat. Immunol. *13*, 753–760.

Wunderlich, P., Glebov, K., Kemmerling, N., Tien, N.T., Neumann, H., and Walter, J. (2013). Sequential proteolytic processing of the triggering receptor expressed on myeloid cells-2 (TREM2) protein by ectodomain shedding and gamma-secretase-dependent intramembranous cleavage. J. Biol. Chem. *288*, 33027–33036.

Yeh, F.L., Wang, Y., Tom, I., Gonzalez, L.C., and Sheng, M. (2016). TREM2 binds to apolipoproteins, including APOE and CLU/APOJ, and thereby facilitates uptake of amyloid-beta by microglia. Neuron *91*, 328–340.

Yuan, P., Condello, C., Keene, C.D., Wang, Y., Bird, T.D., Paul, S.M., Luo, W., Colonna, M., Baddeley, D., and Grutzendler, J. (2016). TREM2 haplodeficiency in mice and humans impairs the microglia barrier function leading to decreased amyloid compaction and severe axonal dystrophy. Neuron *92*, 252–264.

Zhang, H., Xue, C., Shah, R., Bermingham, K., Hinkle, C.C., Li, W., Rodrigues, A., Tabita-Martinez, J., Millar, J.S., Cuchel, M., et al. (2015). Functional analysis and transcriptomic profiling of iPSC-derived macrophages and their application in modeling Mendelian disease. Circ. Res. *117*, 17–28.

Zhang, Y., Sloan, S.A., Clarke, L.E., Caneda, C., Plaza, C.A., Blumenthal, P.D., Vogel, H., Steinberg, G.K., Edwards, M.S., Li, G., et al. (2016). Purification and characterization of progenitor and mature human astrocytes reveals transcriptional and functional differences with mouse. Neuron *89*, 37–53.

Stem Cell Reports, Volume *10*

Supplemental Information

Functional Studies of Missense TREM2 Mutations in Human Stem Cell--Derived Microglia

Philip W. Brownjohn, James Smith, Ravi Solanki, Ebba Lohmann, Henry Houlden, John Hardy, Sabine Dietmann, and Frederick J. Livesey

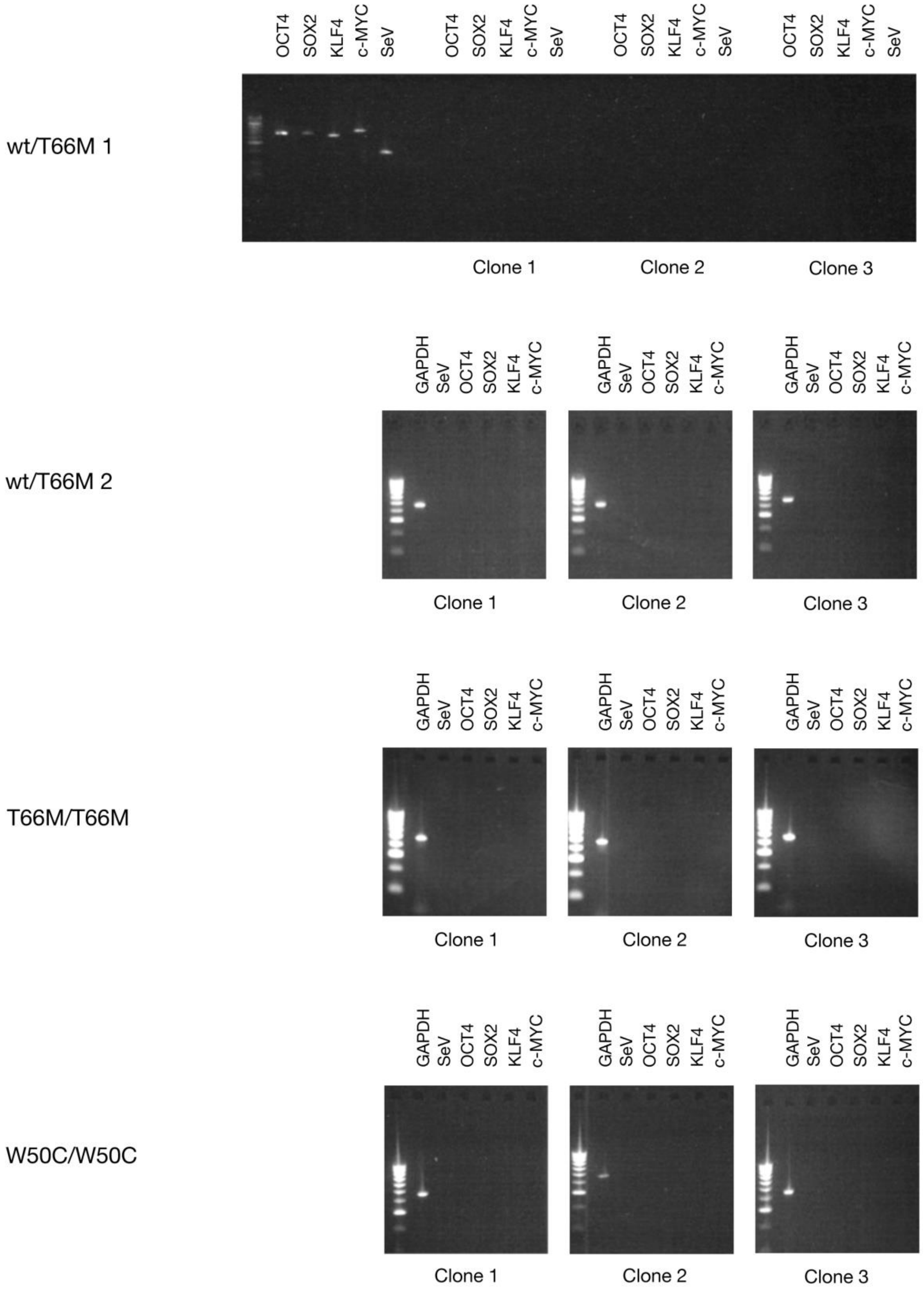
wt/T66M 1
OCT4
SOX2
KLF4
c-MYC
SeV
Clone 1
Clone 2
Clone 3
wt/T66M 2
GAPDH
SeV
OCT4
SOX2
KLF4
c-MYC
Clone 1
Clone 2
Clone 3
T66M/T66M
GAPDH
SeV
OCT4
SOX2
KLF4
c-MYC
Clone 1
Clone 2
Clone 3
W50C/W50C
GAPDH
SeV
OCT4
SOX2
KLF4
c-MYC
Clone 1
Clone 2
Clone 3

Figure S1. Clearance of Sendai virus from reprogrammed iPSCs. (Relates to *TREM2* mutant lines generated for Figures 4 – 6)

Confirmation of clearance of Sendai reprogramming virus from iPSCs of each genotype and donor, assessed by a lack of Sendai virus RNA (SeV) and absent expression of exogenous transgenes (*OCT4*, *SOX2*, *KLF4*, and *c-MYC*). RT-PCR was carried out for each RNA on three representative clones from each iPSC line. Genotypes and starting fibroblast donor are as labelled.

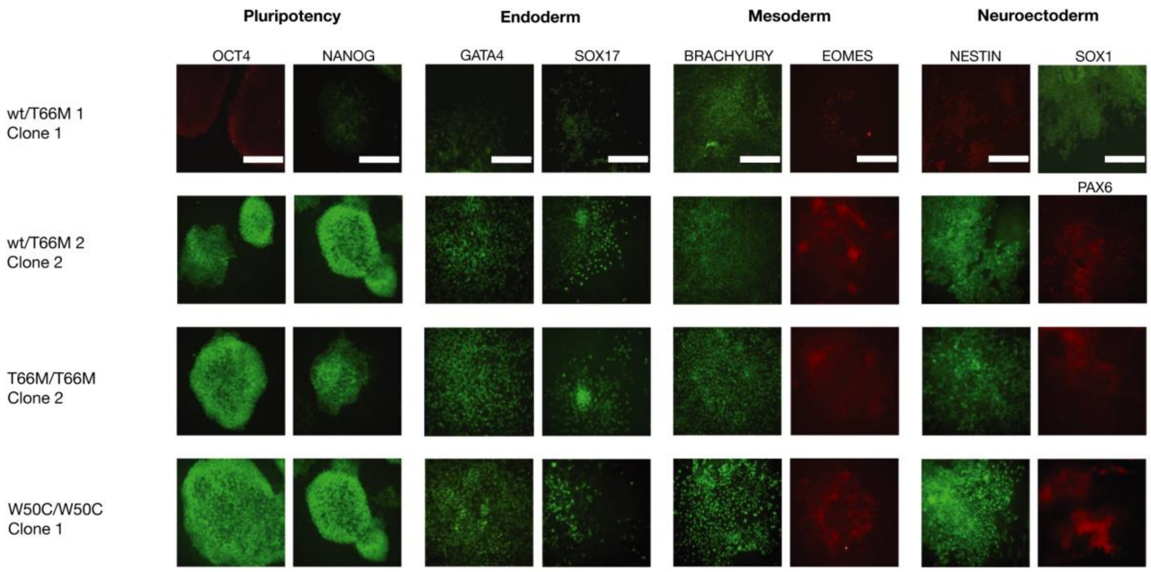

Figure S2. Generation of embryoid bodies and differentiation of a representative iPSC clone from each individual. (Relates to *TREM2* mutant lines generated for Figures 4 – 6)

Expression of pluripotency factors (OCT4, NANOG) was confirmed in starting iPSCs, and expression of markers specific for each of the three major lineages analysed in EBs: endoderm (GATA4, SOX17), mesoderm (BRACHURY, EOMES) and neuroectoderm (NESTIN, SOX1/PAX6). Scale bars represent 400 μm.

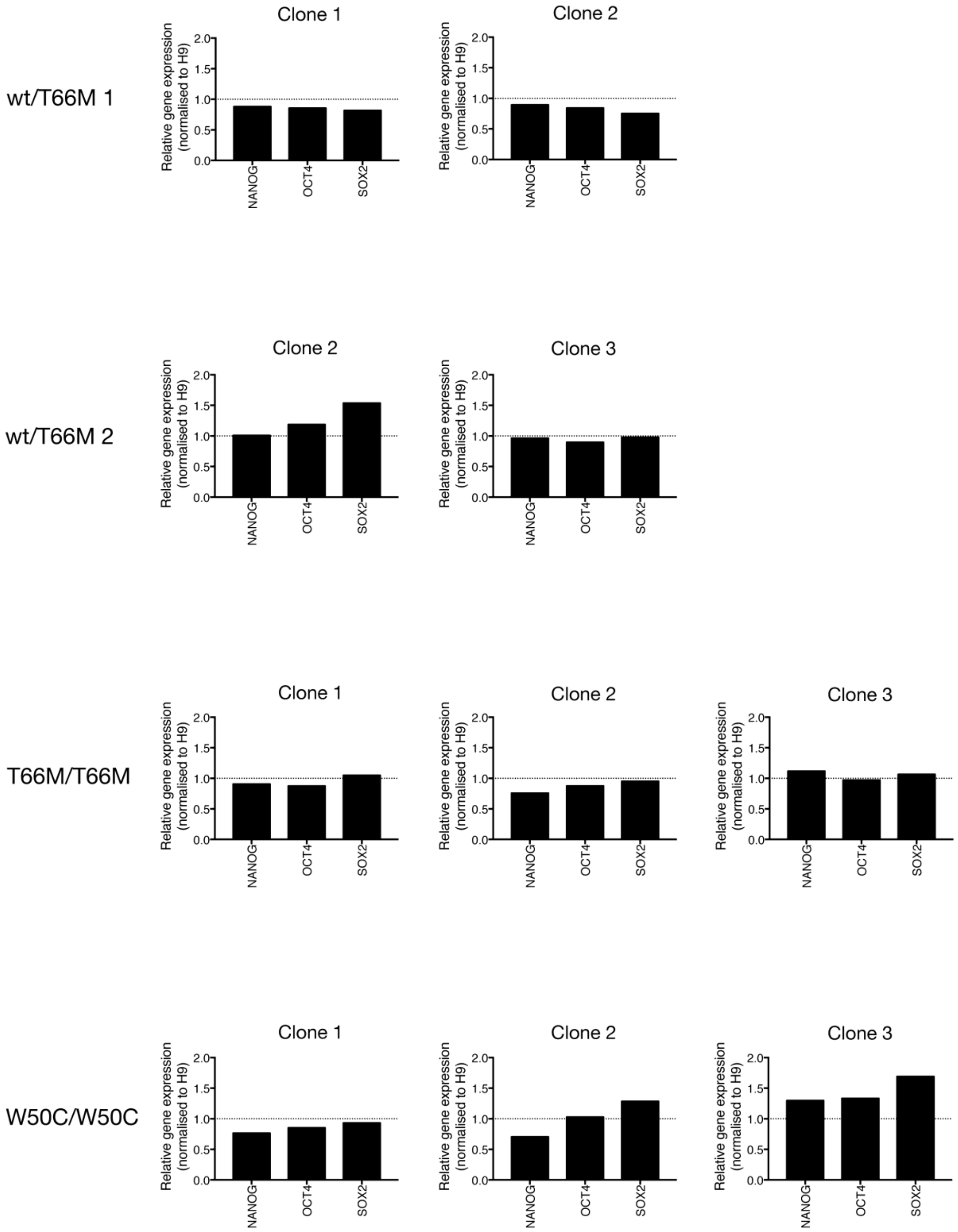

wt/T66M 1
Clone 1
Clone 2
wt/T66M 2
Clone 2
Clone 3
T66M/T66M
Clone 1
Clone 2
Clone 3
W50C/W50C
Clone 1
Clone 2
Clone 3
Relative gene expression (normalised to H9)
0.0
0.5
1.0
1.5
2.0
NANOG
OCT4
SOX2

Figure S3. Confirmation of pluripotency of *TREM2* mutant iPSC lines following adaptation to feeder-free culture. (Relates to *TREM2* mutant lines generated for Figures 4 – 6)

Expression of the pluripotency transcripts *NANOG*, *OCT4* and *SOX2* was confirmed in individual clones from each line using a Nanostring nCounter gene expression array. Gene expression levels were normalised to housekeeping genes, and then to expression levels in the pluripotent embryonic stem cell line H9.

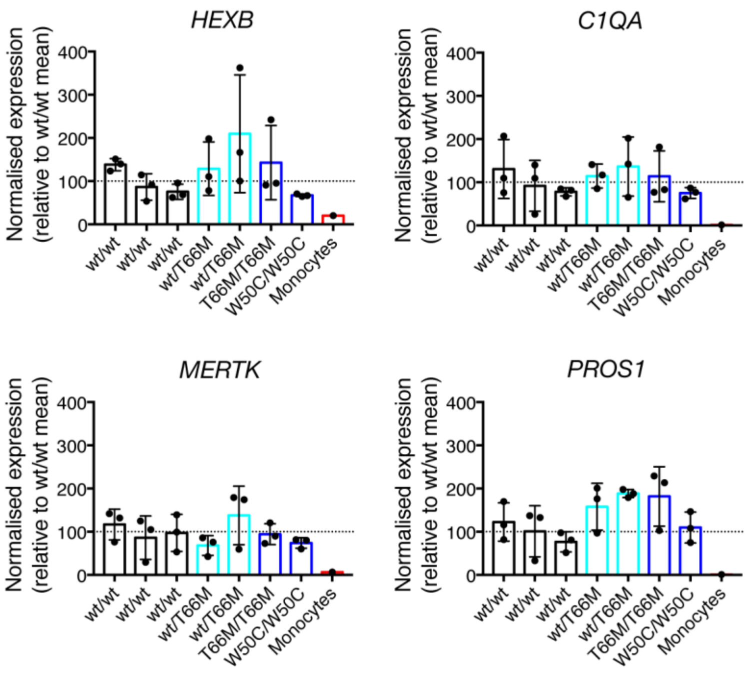

Figure S4. Expression of microglial signature genes in microglia differentiated from wildtype and *TREM2* mutant backgrounds. (Relates to *TREM2* mutant lines generated for Figures 4 – 6)

Microglia differentiated from *TREM2* wildtype (black bars) and heterozygous (cyan bars) and homozygous (dark blue bars) mutant backgrounds express the microglial signature genes *HEXB*, *C1QA*, *MERTK* and *PROS1* as assessed by qPCR. Gene expression levels are shown from three independent differentiations per genetic background, normalised first to the housekeeping genes *ACTB* and *RPS13*, and then to the average expression of all three *TREM2* wildtype backgrounds. As a negative control for these microglia-enriched transcripts, expression levels of each gene are shown for CD14+ peripheral blood monocytes (n = 1). Error bars represent standard deviations.

Hexb	Fscn1	Commd9
Tmem119	Lhfpl2	Adi1
P2ry12	Rcan1	Ndufs3
Lgmn	Cyb5	Mpi
Olfml3	Gpr165	G6pc3
Sgk1	Dnajb4	Dusp7
C1qa	Ctsf	Slc25a4
Plxdc2	CCl2	Tmem258
CCr5	PALDI1	Znf691
Golm1	Cd34	Thrsp
Itgb5	Rps27l	Syngr1
Ltc4s	Gpr155	Tbcb
Bin1	Stau1	Lman1
Lrrc3	Tmem14c	C4orf19
Dynlrb1	COX14	Lap3
Pros1	Upk1b	Ctso
Slc2a5	Itga6	Kiaa0141
Crybb1	FAM212A	Rapgef5
Ecscr	Cxxc5	C9orf69
Cmklr1	Dpm3	Slc39a1
Acp2	Serpinf1	Klhl21
Tanc2	Il1a	Tspan7
Fcgr1A	Tfpi	Stx8
Fcgr1B	Sgce	Arhgap12
Fam102b	Tmem37	Smad1
Snn	Orai1	
Ndufa3	Polr2g	
Ndufc2	Tgfa	
Rtn4rl1	Ubash3b	
Comt	Cryba4	
Etv5	Eif2s1	
Ang	Dctpp1	
LINC00116	Tspan3	
GPR183	P3H2	
Rab3il1	Ndufa7	

Table S1. List of genes upregulated in murine CD45+/TMEM119+ microglia versus CD45+/TMEM119- CNS myeloid cells (Bennett et al., 2016). (Relates to Figure 2)

ACTB_fwd	CACAGAGCCTCGCCTTT
ACTB_rev	GAGCGCGGCGATATCAT
RPS13_fwd	CGAAAGCATCTTGAGAGGAACA
RPS13_rev	TCGAGCCAAACGGTGAATC
HEXB_fwd	GGGAGCATTACGAGGTTTAGAG
HEXB_rev	GGTGGATTCATTGATGGTGAAAG
C1QA_fwd	GGGAAGAAAGGGGAGGCAGG
C1QA_rev	GTTTCCAGAGGGCCCAGGTT
MERTK_fwd	CTTCTCCATGGCCACAGGTT
MERTK_rev	ATACTGAAAAGGTGGGGCGG
PROS1_fwd	AAGAAGCCAGGGAGGTCTTTG
PROS1_rev	ACGTGCAGCAGTGAATAACC

Table S2. Primers used in qPCR. (Relates to Figure S4)

Supplemental Experimental Procedures

Stem cell lines

TREM2 wildtype (wt/wt) cell lines used in this study were AD3 Clone 4 (wt/wt 1), obtained through the IMI StemBANNC consortium, non-demented control Clone 2 (wt/wt 2) (Israel et al., 2012), and the GFP-expressing embryonic stem cell line H9 Cre-LoxP (wt/wt 3) (WiCell).

TREM2 mutation lines were generated from fibroblasts obtained from two donors heterozygous and one patient homozygous for the T66M *TREM2* mutation (2 x wt/T66M; 1 x T66M/T66M), all from the same family, and one patient homozygous for the W50C *TREM2* mutation (W50C/W50C), from skin punch biopsies collected following informed consent and institutional ethics approval. Using non-integrating, four-factor Sendai virus, fibroblasts were reprogrammed through the NIHR Cambridge Biomedical Research Centre (BRC), human Induced Pluripotent Stem Cells core facility. Clones from each genetic background generated for this study were confirmed as free from reprogramming virus by RT-PCR (Figure S1), and pluripotent by differentiation into multiple germ line lineages (Figure S2) and expression of expected pluripotency transcripts (Figure S3). Clones used for primary studies in this paper were: wt/T66M 1 Clone 1; wt/T66M 2 Clone 3; T66M/T66M Clone 1; and W50C/W50C Clone 1.

CytoSNP-850K analysis revealed that, with the exception of wt/T66M donor 2 Clone 3 that was XYY (other clones of this donor untested), all other lines used in this study had expected copy numbers. There was loss of heterozygosity (LoH) on chromosomes 5 (5q13.3 to 5q31.2; 65.1Mb) and 12 (12q13.3 to 12q23.1; 45.5Mb) of wt/T66M donor 2 Clone 3, chromosomes 6 (6p21.2 to 6q22.31; 86.3Mb), 7 (7q11.2 to 7q31.2; 44.9Mb) and 13 (13q14.2 to 13q21.3; 21.4Mb) of all clones derived from patient T66M/T66M, and chromosome 6 (6p22.2 to 6p12.3; 21.4Mb) of all clones derived from patient W50C/W50C. Regions of LoH in patient T66M/T66M, at least in the *TREM2* containing region of Chromosome 6, were previously reported (Guerreiro et al., 2013), and it is hypothesised that this phenomenon precipitated homozygosity of the T66M mutation in this patient. It is likely that this phenomenon also unmasked the recessive W50C mutation in patient W50C/W50C, as the LoH region in this case also encompasses the *TREM2* locus.

Expected *TREM2* mutations were confirmed as present in *TREM2* mutant lines and absent in *TREM2* wildtype lines by Sanger sequencing of exon two of the *TREM2* gene.

Cortical organoid cell culture

Cortical organoids were generated as previously described (Qian et al., 2016) with minor modifications. Briefly, human PSC colonies were dissociated with dispase and cultured overnight in a 10 mL conical tube with Essential 8 medium (day 0). On day 1, colonies were transitioned to ultra-low attachment 6-well plates (Corning) and cultured in Essential 6 medium (Invitrogen) with 2 μM Dorsomorphin (Tocris) and 2 μM A83-01 (Sigma). Media was replaced daily. On day 5-6, half the media was replaced with induction media composed of DMEM:F12 with Glutamax (Invitrogen), 1xN2 Supplement (Invitrogen), 10 μg/mL heparin sulfate (Stem Cell Technologies), 100 U/mL penicillin/streptomycin (Invitrogen), 100 μM Non-essential amino acids (Invitrogen), 4 ng/mL WNT-3A (R&D Systems), 1 μM CHIR99021 (Sigma), and 1 μM SB-431542 (Tocris). On day 7, organoids were embedded in a 2:1 mix of Matrigel (BD Biosciences) and induction media. Induction media was changed daily. On day 14, organoids were mechanically dissociated and cultured in the SpinΩ bioreactor as previously described (Qian et al., 2016).

For microglial infiltration studies, on day 102, cortical organoids were transferred individually to single wells of ultra-low attachment 96-well plates (Corning). Microglia were passaged with Accutase, and resuspended in cortical organoid maturation media (Qian et al., 2016) supplemented with 100 ng/mL IL-34 and 10 ng/mL GM-CSF. 200,000 microglia were added to each organoid. Maturation media was changed every 2 days after the addition of microglia, with IL-34 and GM-CSF removed after the first media change.

Immunofluorescence

After washing in PBS, cell cultures were fixed in 4% (w/vol) paraformaldehyde and blocked with 5% normal donkey serum in 0.3% (vol/vol) Triton-X in TBS before immunofluorescent staining. Unconjugated primary antibodies were directed towards Iba1 (ab5076, Abcam) and TREM2 N-terminal (AF1828, R & D Systems), and PE-conjugated primary antibody was directed towards CD45 (21810454S, Immunotools). Secondary antibodies were Alexa Fluor-conjugated (Thermo Fisher Scientific). For non-permeabilised TREM2 staining, Triton-X was omitted from all blocking and washing steps (Kleinberger et al., 2014).

Cortical organoids and cortical organoid/microglia co-cultures were fixed in 4% (w/vol) paraformaldehyde for 30 minutes at room temperature and cryoprotected with 30% sucrose (w/vol) in PBS overnight. Organoids were embedded in OCT and 12 μm sections generated. Sections were blocked in 5% normal donkey serum in TBS prior to immunofluorescent staining. Primary antibodies were directed to TBR1 (ab31940, Abcam) and SATB2 (ab51502, Abcam), and secondary antibodies were Alexa Fluor-conjugated (Thermo Fisher Scientific).

Imaging of immunocytochemistry and immunohistochemistry was performed on an inverted confocal microscope (Leica TCS SP8 X white light laser) and an Opera Phenix high content imaging system (PerkinElmer), and images analysed with Volocity (PerkinElmer), or Harmony (PerkinElmer). After transfer to 96 well imaging plates (Ibidi), cortical organoid/microglia co-cultures were imaged live with a Marianas inverted 2-photon microscope (3i).

Microglial differentiation efficiency, as determined by labeling with Iba1, CD45 and TREM2, was quantified using the Opera Phenix. Cells were counted as label positive when mean fluorescent intensity exceeded background levels, which was determined by omission of primary antibody (for unconjugated primary antibodies Iba1 and TREM2) or non-specific binding by IgG control (for conjugated primary antibody CD45). Iba1 and CD45 labeling was quantified in microglia differentiated from *TREM2* wildtype and *TREM2* heterozygous and homozygous mutant lines, whereas TREM2 labeling was quantified in *TREM2* wildtype and heterozygous mutants only.

Transcriptome analysis by RNA-Seq

To obtain PMP and microglia RNA samples, three independent differentiations of two genetic backgrounds (n = 5 PMP, n = 6 microglia) were performed. After two PBS washes, RNA was extracted using the RNeasy micro kit (Qiagen) as per manufacturer's instructions, with RIN confirmed as greater than 8.5 in all samples. cDNA libraries were prepared with 250ng of input RNA using a TruSeq Stranded Total RNA kit (RS-122-2201, Illumina), and subjected to 50 bp single end sequencing. RNA-seq reads were quality-trimmed using *Trim Galore!,* and mapped to the human reference genome (GRCh37/hg38) using *TopHat2* with the parameters "--max-multihits 1 --read-mismatch 2 --b2-sensitive". ENSEMBL (release 86) gene models were used to guide alignments with the "–GTF" option. Read counts for genes were obtained using *featureCounts* with the parameters "-p –s 1 –O --minOverlap 10 –B –C". Read counts were normalized, and the statistical significance of differential expression was assessed using the R *Bioconductor DESeq2* package. Gene counts - normalized by *DESeq2* size factors - were subsequently normalized by their effective transcript length/1000, and log2-transformed. Effective transcript lengths were obtained with *featureCounts.* Principal Component Analysis (PCA) was performed using singular value decomposition on scaled expression values with the R *princomp()* function. For FPKM counts, independent differentiations of stem-cell derived microglia were averaged as technical replicates for each genetic background.

Reported gene expression datasets were downloaded from Gene Expression Omnibus (www.ncbi.nlm.nih.gov/geo) under accession numbers GSE73721 (Zhang et al., 2016) (*ex vivo* brain CD45+ microglia/macrophages), GSE55536 (Zhang et al., 2015) (monocyte-derived macrophages), and GSE89189 (Abud et al., 2017) (CD14+/CD16- monocytes, dendritic cells, alternative iPSC-derived microglia, *in vitro* microglia and fetal microglia); or from dbGaP (https://www.ncbi.nlm.nih.gov/gap) under accession number phs001373.v1.p1 (Gosselin et al., 2017) (*ex vivo* and *in vitro* microglia), and processed in the same way. For samples from (Gosselin et al., 2017), only samples from individuals in which matched *ex vivo* and 7 or 10 day *in vitro* cultured (DMEM/F12 + FBS + IL-34) samples were available were used for comparisons.

Gene expression analysis (Nanostring nCounter and RT-PCR)

Expression of the pluripotency genes *NANOG*, *OCT4* and *SOX2* was confirmed in all iPSC lines on the Nanostring nCounter platform using the human stem cell panel of genes, after initial normalisation to housekeeping genes and then expression levels of each transcript in the pluripotent embryonic stem cell line H9 (WiCell).

Semi-quantitative RT-PCR was performed on total RNA from three independent microglial differentiations of each stem cell line used for primary experiments in this study, and CD14+ peripheral blood monocytes (SER-CD14-F, Zenbio). After PBS wash, cells were lysed in RNAlater buffer, and total RNA extracted with the RNeasy mini kit (Qiagen). cDNA was reverse transcribed from RNA with a high-capacity cDNA reverse transcription kit (4368814, Applied Biosystems), using a 1:1 mix of random primers and oligo(dT). qPCR was performed on the StepOnePlus real time PCR system using SYBR Green JumpStart Taq Readymix (S4438, Sigma). Genes of interest were normalised to the geomean of the housekeeping genes *ACTB* and *RPS13*. Primer sequences are supplied in Table S2.

Protein analysis by Immunoblotting

After PBS wash, cells were lysed in 4x LDS sample buffer containing 2 mM DTT. After boiling, cell lysates were subjected to SDS-PAGE and immunoblotting. Primary antibodies used were directed towards calnexin (ADI-SPA-860, Enzo Life Sciences), TREM2 N-terminal (AF1828, R & D Systems) and TREM2 C-terminal (91068, Cell Signalling Technology). Immunoblot detection was performed with Licor secondary antibodies, and analysis performed using the Odyssey Infrared Imaging System.

Lipopolysaccharide challenge

Lipopolysaccharide (LPS) from E. coli (O55:B5, Sigma) was resuspended in PBS before further dilution in microglia media for use in assays. Microglia were treated with LPS-containing microglia media for 6 or 24 hours, with or without co-administration with IFNγ (20 ng/mL), before extracellular media was collected, spun at 800 X G for 3 min to remove contaminating cells, and resultant supernatant stored at -20°C. Microglial supernatant was analysed with multiplexed ELISA (Meso Scale Diagnostics) allowing parallel assessment of secreted IL-1β, TNFα, and IL-6 protein levels from the same sample. For LPS experiments comparing *TREM2* genotypes, released cytokine levels were normalised to cell number measured at baseline on the day of treatment using the Opera Phenix imaging system (PerkinElmer). All treatments were performed in duplicate or triplicate wells.

Phagocytosis and internalisation assays

To measure phagocytosis of bacterial particles, live imaging was performed on microglia exposed to 50 μg/mL pHrodo red conjugated *E. coli* bioparticles (P35361, Invitrogen). To assess the potentially confounding effect of serum on phagocytosis, bioparticles were presented either in standard serum-containing microglia media, or serum-absent microglia media following overnight serum starvation (18 hours exposure to 1% FBS-containing microglia media). As a positive control for reduced phagocytosis, microglia were pre-treated (for 45 min) and then co-treated with 10 μM of the actin polymerisation inhibitor cytochalasin D. As pHrodo labelled dyes are pH sensitive, and fluoresce preferentially in acidic environments such as endosomes and lysosomes, live imaging could be performed in culture media over two or three hours with a high temporal resolution (every 6 min) in order to assess phagocytosis kinetics.

To measure uptake of acetylated low-density lipoprotein (AcLDL), microglia were treated with 5 μg/mL DiI-complexed human AcLDL (L3484, Invitrogen) in microglia media. Thirty minutes or three hours after exposure, AcLDL containing media was removed, and replaced with serum-free RPMI, containing 0.02% trypan blue in order to quench extracellular fluorescence, before immediate imaging.

Live imaging of pHrodo bioparticle uptake and fixed imaging of DiI-complexed AcLDL was performed on the Opera Phenix imaging platform (PerkinElmer), with uptake analysis of both substrates performed with custom parameters within Harmony (PerkinElmer). For pHrodo bioparticle uptake, area of pHrodo signal in the fluorescent channel was measured and calculated as a proportion of total cell area measured in the digital phase

contrast (DPC) channel, while for AcLDL uptake, average intensity across each well of mean DiI signal per cell was calculated.

SUPPLEMENTARY REFERENCES

Abud, E.M., Ramirez, R.N., Martinez, E.S., Healy, L.M., Nguyen, C.H.H., Newman, S.A., Yeromin, A.V., Scarfone, V.M., Marsh, S.E., Fimbres, C., *et al.* (2017). iPSC-Derived Human Microglia-like Cells to Study Neurological Diseases. Neuron *94*, 278-293 e279.

Bennett, M.L., Bennett, F.C., Liddelow, S.A., Ajami, B., Zamanian, J.L., Fernhoff, N.B., Mulinyawe, S.B., Bohlen, C.J., Adil, A., Tucker, A., *et al.* (2016). New tools for studying microglia in the mouse and human CNS. Proceedings of the National Academy of Sciences of the United States of America *113*, E1738-1746.

Gosselin, D., Skola, D., Coufal, N.G., Holtman, I.R., Schlachetzki, J.C.M., Sajti, E., Jaeger, B.N., O'Connor, C., Fitzpatrick, C., Pasillas, M.P., *et al.* (2017). An environment-dependent transcriptional network specifies human microglia identity. Science *356*.

Guerreiro, R.J., Lohmann, E., Bras, J.M., Gibbs, J.R., Rohrer, J.D., Gurunlian, N., Dursun, B., Bilgic, B., Hanagasi, H., Gurvit, H., *et al.* (2013). Using exome sequencing to reveal mutations in TREM2 presenting as a frontotemporal dementia-like syndrome without bone involvement. JAMA Neurol *70*, 78-84.

Israel, M.A., Yuan, S.H., Bardy, C., Reyna, S.M., Mu, Y., Herrera, C., Hefferan, M.P., Van Gorp, S., Nazor, K.L., Boscolo, F.S., *et al.* (2012). Probing sporadic and familial Alzheimer's disease using induced pluripotent stem cells. Nature *482*, 216-220.

Kleinberger, G., Yamanishi, Y., Suarez-Calvet, M., Czirr, E., Lohmann, E., Cuyvers, E., Struyfs, H., Pettkus, N., Wenninger-Weinzierl, A., Mazaheri, F., *et al.* (2014). TREM2 mutations implicated in neurodegeneration impair cell surface transport and phagocytosis. Science translational medicine *6*, 243ra286.

Qian, X., Nguyen, H.N., Song, M.M., Hadiono, C., Ogden, S.C., Hammack, C., Yao, B., Hamersky, G.R., Jacob, F., Zhong, C., *et al.* (2016). Brain-Region-Specific Organoids Using Mini-bioreactors for Modeling ZIKV Exposure. Cell *165*, 1238-1254.

Zhang, H., Xue, C., Shah, R., Bermingham, K., Hinkle, C.C., Li, W., Rodrigues, A., Tabita-Martinez, J., Millar, J.S., Cuchel, M., *et al.* (2015). Functional analysis and transcriptomic profiling of iPSC-derived macrophages and their application in modeling Mendelian disease. Circ Res *117*, 17-28.

Zhang, Y., Sloan, S.A., Clarke, L.E., Caneda, C., Plaza, C.A., Blumenthal, P.D., Vogel, H., Steinberg, G.K., Edwards, M.S., Li, G., *et al.* (2016). Purification and Characterization of Progenitor and Mature Human Astrocytes Reveals Transcriptional and Functional Differences with Mouse. Neuron *89*, 37-53.
